# Bone Accumulation by Leopards in the Late Pleistocene in the Moncayo Massif (Zaragoza, NE Spain)

**DOI:** 10.1371/journal.pone.0092144

**Published:** 2014-03-18

**Authors:** Víctor Sauqué, Raquel Rabal-Garcés, Cristina Sola-Almagro, Gloria Cuenca-Bescós

**Affiliations:** 1 Grupo Aragosaurus-IUCA, Paleontología, Facultad de Ciencias, Universidad de Zaragoza, Zaragoza, Spain; 2 Ciencias de la antigüedad, Facultad de Filosofía y Letras, Universidad de Zaragoza, Zaragoza, Spain; University of Florence, Italy

## Abstract

Eating habits of *Panthera pardus* are well known. When there are caves in its territory, prey accumulates inside them. This helps to prevent its kill from being stolen by other predators like hyenas. Although the leopard is an accumulator of bones in caves, few studies have been conducted on existing lairs. There are, however, examples of fossil vertebrate sites whose main collecting agent is the leopard. During the Late Pleistocene, the leopard was a common carnivore in European faunal associations. Here we present a new locality of Quaternary mammals with a scarce human presence, the cave of Los Rincones (province of Zaragoza, Spain); we show the leopard to be the main accumulator of the bones in the cave, while there are no interactions between humans and leopards. For this purpose, a taphonomic analysis is performed on different bone-layers of the cave.

## Introduction

The leopard *Panthera pardus* is the large feline with the greatest present distribution area, covering most of Africa and Asia [1], [2], [3], [4]. This exceptionally broad distribution is due to its great potential for adaptation, displaying a great variety of behaviours that depend on the habitat it occupies. The leopard is territorial and a solitary hunter that uses an ambush technique [1], [2], [3], [4], [5]. For this reason, it is forced to protect its kill from other social predators such as hyenas or canids. To achieve this, leopards have two different strategies at their disposal: in open areas such as the savannah, they haul their prey up into a tree [6,7,8,910], whereas in areas where there are caves they prefer to transport and accumulate their prey inside them references in de Ruiter & Berger [11]. Even though the leopard is a potential accumulator of bones in caves, only a few studies of present-day dens have been carried out [8], [12], [13], [14]–[15], and they have been practically excluded from the formation of sites [16]. However, there are various examples of sites with fossil vertebrates accumulated by leopards, specifically the well-known sites with human fossils, Swartkrans Members 1 and 2 [8], [16], [17], [18].

During the Late Pleistocene the leopard was a common element in the faunal association of Europe, but mainly recorded on the basis of scarce and fragmentary dentognathic material preventing a good knowledge of its behaviour. Nevertheless, fragmentery material is found in more than 100 sites (see references in [19]) prior to its disappearance around the Late Pleistocene-Holocene boundary. Its final appearance is recorded in the north of Spain [19]. In spite of its broad distribution, there are only a very few references to fossil sites at which the role of the leopard as an accumulator is an important one. At Gabasa 1 the leopard is a taphonomic agent, whose importance is less than that of the hyena or wolf [20], [21]; at La Caune de l’Arago, in levels MNO of CM1, the leopard is one possible accumulator among other carnivores [22], [23]; at Baumann’s Cave, there are at least a tooth and metacarpus of subadult ibex that might refer to a leopard lair situation at the former second entrance [24]. The problem of European leopard lair sites is the abundant overlap with human camp and other carnivore dens in rock shelter positions [24].There are only two possibles sites which the leopard plays a major role Amalda VII and Allekoaitze, both are situated in the North of Spain geographically close to Los Rincones. In the accumulation Amalda VII leopard and lynx are the principal accumulators of small-sized ungulates [25], [26], and in Allekoaize leopard seems to be the main accumulator of the Ibex remains [24].

The aim of the present paper is to present the cave of Los Rincones (Zaragoza, Spain), a site with a human presence (lithic industry and marks on bones) and a high percentage of carnivores that have contributed to its formation. The main objective of the paper is to ascertain the identity of the main accumulator of this bone accumulation. To this end, we undertake a taxonomic study of the taphocoenosis, and a taphonomic analysis is carried out.

### The cave of Los Rincones

The palaeontological site of the cave of Los Rincones was discovered by members of CEA (Centro de Espeleología de Aragón) in 2005 while they were mapping the cave. The cave of Los Rincones is described by the CEA members who unclogged a small entrance led to enter an old gallery which preserves the bones in the position that they were accumulated [27]. The presence of bones, especially a complete skull of brown bear in the cave, was reported to Gloria Cuenca, who visited the cave in 2006 in company of Juan Luis Arsuaga, Milagros Algaba and members of CEA. During this visit we observed that there were indeed many bone remains scattered in the surface of Ursus Gallery and Leopard Gallery. Subsequently we conducted several geological surveys during 2009 and 2010 to collect the stratigraphic, taphonomic, and cartographic-photographic data. The cave of Los Rincones is situated in the Sierra del Moncayo, which is located in the central part of the Iberian Range in the north of Spain. As a result of the altitude of the Sierra and its geographical situation between the river basins of the Duero and the Ebro, the area receives a substantial hydrological input generated by the Atlantic frontal systems.

**Figure 1:**
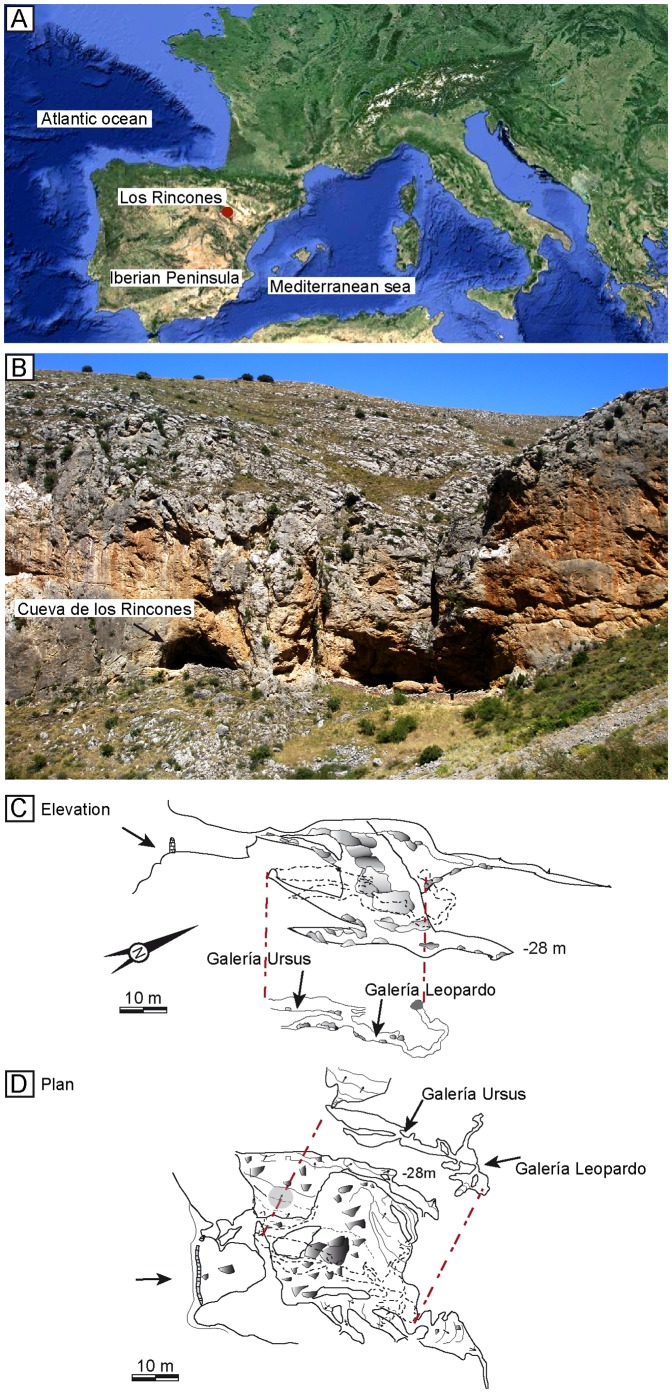
Geographical location and topography of the Los Rincones cave. A, geographical location of los Rincones cave. B, panoramic view of the Los Rincones ravine. C and D elevation and plan views of the Los Rincones cave.

The cave is situated at the head of the ravine of Los Rincones, in the municipality of Purujosa (Zaragoza). The mouth of the cave opens at an altitude of 1010 m. It is a complex cave, consisting of various chambers and galleries located at different heights (Fig 1).

**Figure 2:**
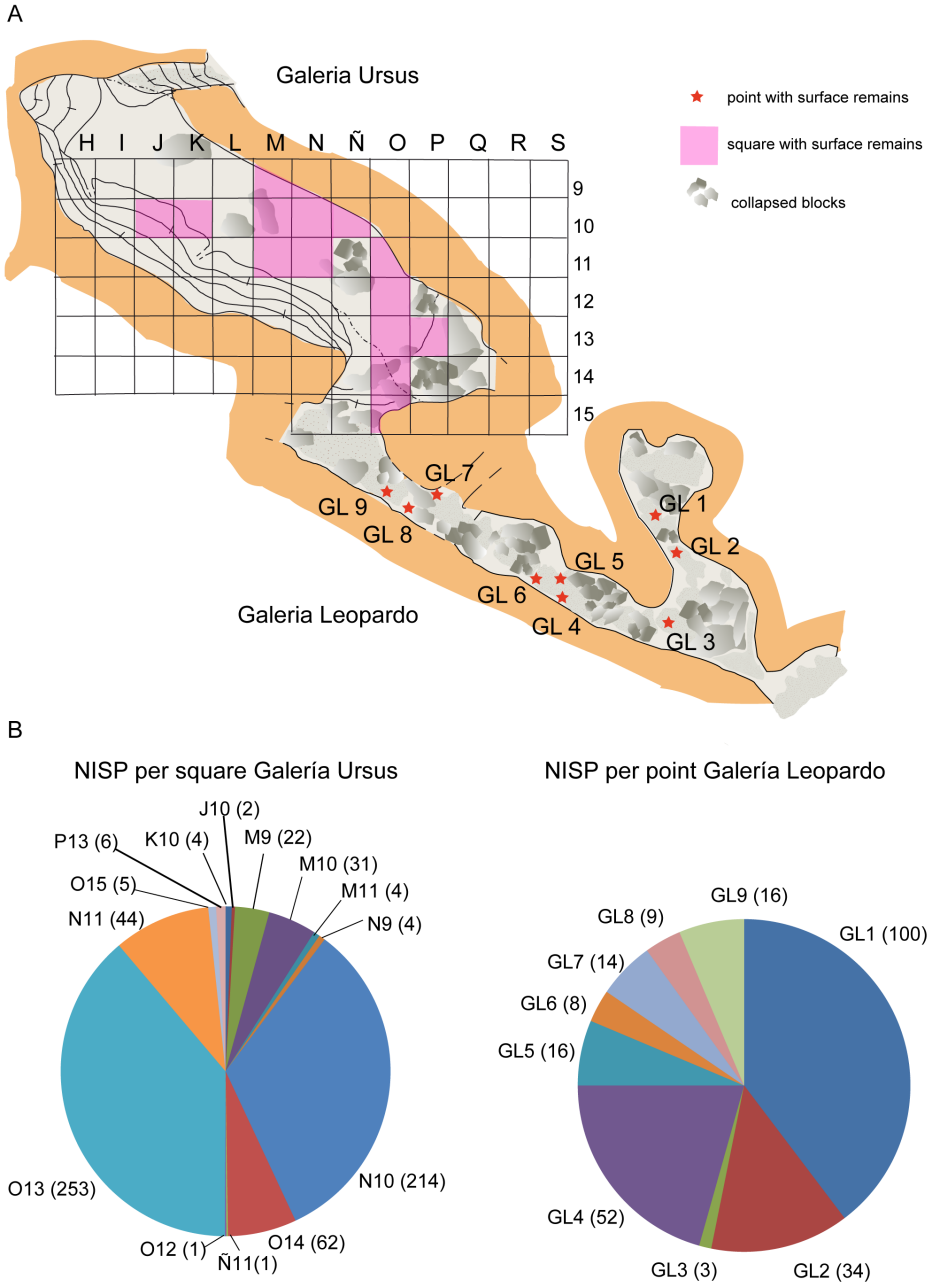
Plan view of Los Rincones with grid and NISP in each part. A. plan view of the Los Rincones with the grid in Galería Ursus and collecting points in Galería Leopardo B. NISP per square in Galería Ursus and NISP per point in Galería Leopardo.

The fossil remains under study in the present paper were provisionally housed at the University of Zaragoza and has been given the field specimen number: Ri10 J10, 112 to 113; Ri10 K10, 109 to 111; Ri10 M9, 1 to 19; Ri10 M10, 1 to 15; Ri10 M11, 1–4; Ri10 N10, 1 to 316; Ri10, N11,1 to 45; Ri10 O12, 1 to 2; Ri10 O13, 1 to 291; Ri10 O14, 1 to 54; Ri10 P13, 1–7; Ri10 GL1, 1 to 128, Ri10 GL2 1 to 30; Ri10 GL3, 1 to 9; Ri10 GL4, 1 to 56; Ri10 GL5, 1 to 21; Ri10 GL6, 1 to 11; Ri10 GL7, 1 to 21; Ri10 GL9, 1 to 18. The fossil remains were discovered during preliminary prospections of the cave of Los Rincones under the authorization of the Government of Aragon and Parque Natural del Moncayo. The fossils were collecting at the surface of both galleries the “*Ursus* Gallery” (GU) and the “Leopard Gallery” (GL) (Fig 2). In fact, both galleries are passages that run between blocks produced by the collapse of the cave’s walls and ceiling prior to the deposition of sediment, which occurred subsequently and covered the gaps and surfaces between the blocks. The galleries are connected by a series of passages between the blocks [27] (Fig. 1, 2). A collection of the surface remains was undertaken in the GU in order to prevent the bones remaining exposed. To this end, the surface was divided into squares measuring one square metre each. Due to the narrowness and the collapsed blocks in GL, it was not possible to stablish a square so we put a number in the different places of this gallery where we collected bones (Fig 2). The faunal composition and taphonomic alteration is similar in both galleries. In some cases, bone fragments from the same specimen are found in the two galleries. The bony remains from the cave of Los Rincones have made it possible to describe a diverse fauna consisting of large and small mammals [19]. The large-mammal species identified so far in Los Rincones are *Ursus arctos, Canis lupus, Panthera pardus*, *Lynx* sp. (probably the Iberian lynx, *Lynx pardinus*, though it is so poorly represented that we prefer to let it in open nomenclature), *Cervus elaphus, Capreolus capreolus, Capra pyrenaica, Rupicapra pyrenaica,* Bos/Bison sp., *Equus hydruntinus,* and *E. ferus*.

The faunal association suggests a Late Pleistocene age. Unfortunately, we sent samples to Beta Analytics but we could not obtain radiometric ages by the ^14^C method due to lack of collagen in the bones. Likewise, the rodents found in the upper part of the sedimentary cone that closes the original entrance to the cave above the “*Ursus* Gallery” (*Microtus* spp., *Iberomys cabrerae* and *Pliomys lenki)* point to the Late Pleistocene. Specifically, *P. lenki* disappears towards the end of the second third of the Late Pleistocene in the centre of the Iberian Peninsula, where it is found in sites with Mousterian industry [19], [28]. During the early stages of cleaning the gallery, a piece of Mousterian industry was also discovered in the cave of Los Rincones.

## Materials and Methods

The identifiable remains and splinters greater than 4 cm in size have been studied. For the taxonomic identification the following authors were followed: Pales and Lambert [29], Walker [30], Torres [31] Fernandez [32] and Eisenmann [33], and comparisons were made with the reference collections of the University of Zaragoza (UZ) and the Pyrenean Institute of Ecology (Instituto Pirenaico de Ecología, IPE). To evaluate the skeletal representation in the assembly from Los Rincones, the number of remains (NR), the number of identified specimens (NISP), the minimum number of elements (MNE), the minimum number of individuals (MNI) and the skeletal survival rate (%Surv) were used, which were calculated in accordance with Brain [8] and Lyman [34]. To calculate the MNI, the teeth were used, because they are the most common anatomical element, and the degree of eruption and dental wear were also taken into consideration. The %Surv of an element is the ratio between the number of elements recovered and the number of elements expected. It is calculated using the formula %Surv  =  MNE *100/number of these elements in the skeleton * MNI. The bones that could not be assigned to a taxon were included in weight-based categories in accordance with the criteria proposed by Bunn [35], modified by Díez *et al*. [36] (Table 1).

**Table 1:** Criteria used for the classification of unidentified remains from Los Rincones assemblage.

Bunn (1986)			Los Rincones		
Weight sizes	Weight range		Weight sizes	Weight range (Kg)	Taxa and age
	Pounds	Kg			
1	< 50	< 22.65	very small size	< 20	neonatal *Ursus arctos*
					juvenile *Capra pyrenaica*
					adult *Testudo hermanni*
2	50–250	22.65 – 113.25	small size	20–100	adult *Canis lupus*
					adult *Lynx* sp.
					adult *Panthera pardus*
					adult *Rupicapra pyrenaica*
					adult *Capreolus capreolus*
					adult *Capra pyrenaica*
3A	250–450	113.25 – 203.85	middle size	100–300	adult *Cervus elaphus*
3B	450–750	203.85 – 339.75			adult *Ursus arctos*
4	750–2000	339.75 – 996	large size	300–1000	adult *Equus hydruntinus*
					adult *Equus ferus*
					adult Bos/Bison sp.

In general, to determine the age of death of the individuals the dental replacement and degree of eruption were used [37], [38], as well as the fusion of the epiphyses in long bones [39]. More specifically, for *C. pyrenaica* we follow Pérez-Ripoll [40] and Vigal & Machordom [41]; for *C. capreolus* we follow Tomé & Vigne [42]; for *R. pyrenaica* we follow Pérez-Barbería [43]; and for *C. elaphus* we follow Aitken [44], Mariezkurrena [45], Azorit *et al*. [46] and D’Errico & Vanhaeren [47].

As far as anthropic markings are concerned, we differentiate two main types: those that are produced when bones are broken and cut marks. Within the first category, we distinguish percussion notches, impact flakes, percussion pits and peeling [48], [49]; within the category of cut marks, incisions, scrapes and chopmarks have been distinguished [50], [51], [52], [52,54].

Various types of marks produced by carnivore teeth have been differentiated (pits, punctures, grooves, furrowing, crenulated edges and impact points), according to the definitions by Haynes [55], [56],and Sala [57]. The measurements were taken with an electronic digital calliper. To identify the marks made by the carnivores, they were compared with the data provided in the papers by Delaney-Rivera *et al*. [58], Domínguez-Rodrigo & Piqueras [59], Saladié *et al*. [60], Rabal-Garcés *et al.* [61] and Rabal-Garcés [62].

To ascertain whether the breakage of the bones occurred in fresh bone, straight after the animal’s death or a certain time after its burial, as well as the possible causes of the breakage, we follow the criteria proposed by Villa and Mahieu [63]. This method takes into account the delineation (longitudinal, transverse or curved), the angle (oblique, straight or mixed), and the type of edge of the fractures presented by long bones more than 4cm in length, which can be irregular or smooth. In addition, account is taken of the breakage index, which refers to the portion of the diaphysis preserved in relation to both the total length and circumference of the bone. The breakage indices that refer to the length of the diaphysis are L1 (preserved length < ¼ of the total length), L2 (preserved length between ¼ and ½ of the total length), L3 (preserved length between ½ and ¾ of the total length) and L4 (preserved length > ¾ of the total length). The breakage indices for the circumference are C1 (preserved circumference < ½ of the total circumference), C2 (preserved circumference > ½ of the total circumference) and C3 (the circumference is complete or almost complete, at least in some part). To get a better idea of this process of fragmentation, GL was divided into two areas: GL 1–3 is further away from GU, located at a lower level than the others, so it is the area where the remains have undergone the greatest transportation; GL 4–9 is located in an intermediate area between GU and GL 1–3, with length and circumference values between those of GU and GL 1–3.

To establish the origin of the accumulation of bone remains in the cave of Los Rincones we follow the criteria used by Cruz-Uribe [64] for distinguishing accumulations produced by carnivores from anthropic accumulations. We also take into account the papers by [65], [66] that revised these criteria. Moreover, in identifying the accumulating agent, we follow [15], [57], [67], [68], [69].

The degree to which the abundance of species in a fossil association reflects the past community has been studied by Damuth [70]. The relation that exists between body weight and the abundance of these species is an indicator of the real abundance in natural communities. The graphic representation of logA (abundance) versus logBW (body weight) for each of the species, together with the slope of the regression line, allows us to determine whether the abundance of the fossil species represents real abundance in the assumed fossil community, i.e. if the slope falls within the range from –0.8 to –1.3. The present paper uses Damuth’s method to verify the representation of the prey species in the association of Los Rincones.

## Results

**Figure 3.%:**
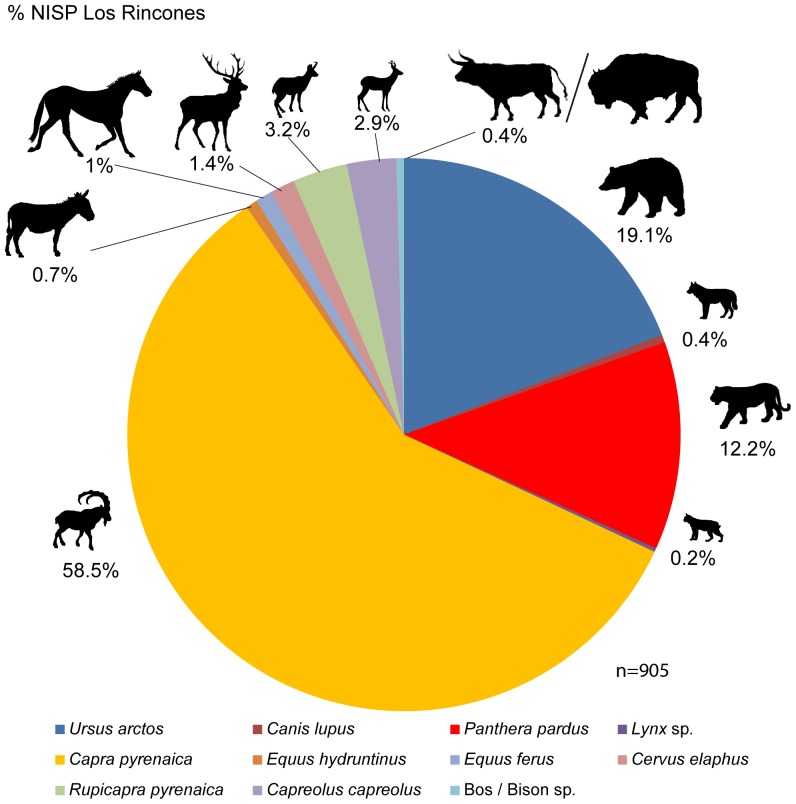
NISP from Los Rincones, n = 905.

At the site of Los Rincones, 1443 remains of fossil bones have been recovered on the surface of Ursus Gallery and Leopard Gallery in diferent places, with a distribution of the remains is not homogeneus [Fig. 2], have been taxonomically identified 905 remains and 318 of which have been assigned to the various size categories. Further, 220 fossils larger than 4cm have been recovered that remain unclassified either taxonomically or within a size category. The MNE is 905. The most frequently represented elements are teeth (233), phalanges (130), vertebrae (102), carpals/tarsals (96),metapodials (88) and ribs (70). The long bones show an analogous representation, comprising (in order of decreasing frequency) humeri (38), radiuses (29), tibiae (28), femora (21) and ulnae (15). Complete or fragmented crania are less represented (18), as are mandibles (16), pelvises (9) and scapulae (12). Most of the taxonomically assigned remains belong to *C. pyrenaica* (528), followed by *U. arctos* (173) and *P. pardus* (110) (Fig 3). As far as the elements classified by size are concerned, small-sized elements are particularly prominent (298). The sum of the three most represented taxa, together with the smaller-sized elements, represents 73.25% of the specimens. The MNI is 46 (Table 2).

**Table 2:** NR, NISP, MNE, MNI by taxa and size categories from Los Rincones faunal assemblage.

						MNI by ages			
	NR	NISP	MNE	MNI	neo.	juvenil	sub ad.	ad	sen.
Ursus arctos	173	173	141	8	1	1	3	3	
Canis lupus	4	4	4	1				1	
Panthera pardus	110	110	97	4				4	
Lynx sp.	2	2	2	1					
Capra pyrenaica	528	528	437	20	1	3	7	5	4
Equus hydruntinus	2	2	2	1				1	
Equus ferus	10	10	10	2				2	
Cervus elaphus	13	13	13	2				3	
Rupicapra pyrenaica	29	29	29	3				3	
Capreolus capreolus	26	26	23	2				2	
Bos/Bison sp.	4	4	4	1				1	
Testudo hermanni	1	1	1	1					
Middle size	5		4						
Small size	298		121						
Very small size	16		15						
Unidentified (> 4cm)	219								
Total	1443	905	905	46	2	4	10	25	4

### Minimum number of individuals

#### Carnivores

**Figure 4:**
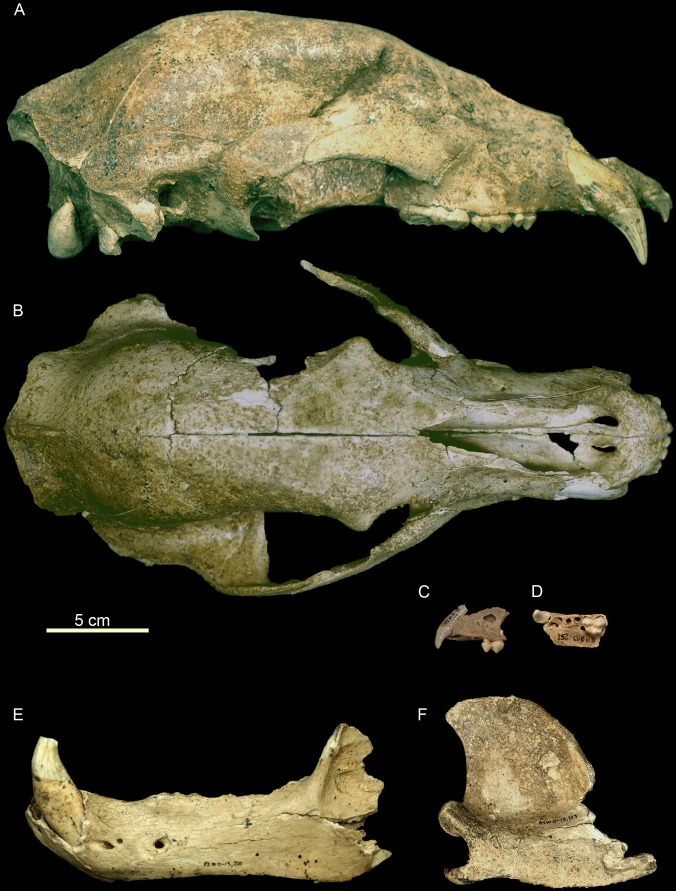
Cranial remains of *Ursus arctos* from the Late Pleistocene of Los Rincones. Cranial remains of *Ursus arctos* from the Late Pleistocene of Los Rincones. A skull of an adult Ri10/O13/34. Left maxilla of a juvenile. Ri10/O13/252, Ri10/O13/175. Right mandible of an adult Ri10/O13/217. Left mandible with canine of an adult Ri10/O13/200.

**Figure 5:**
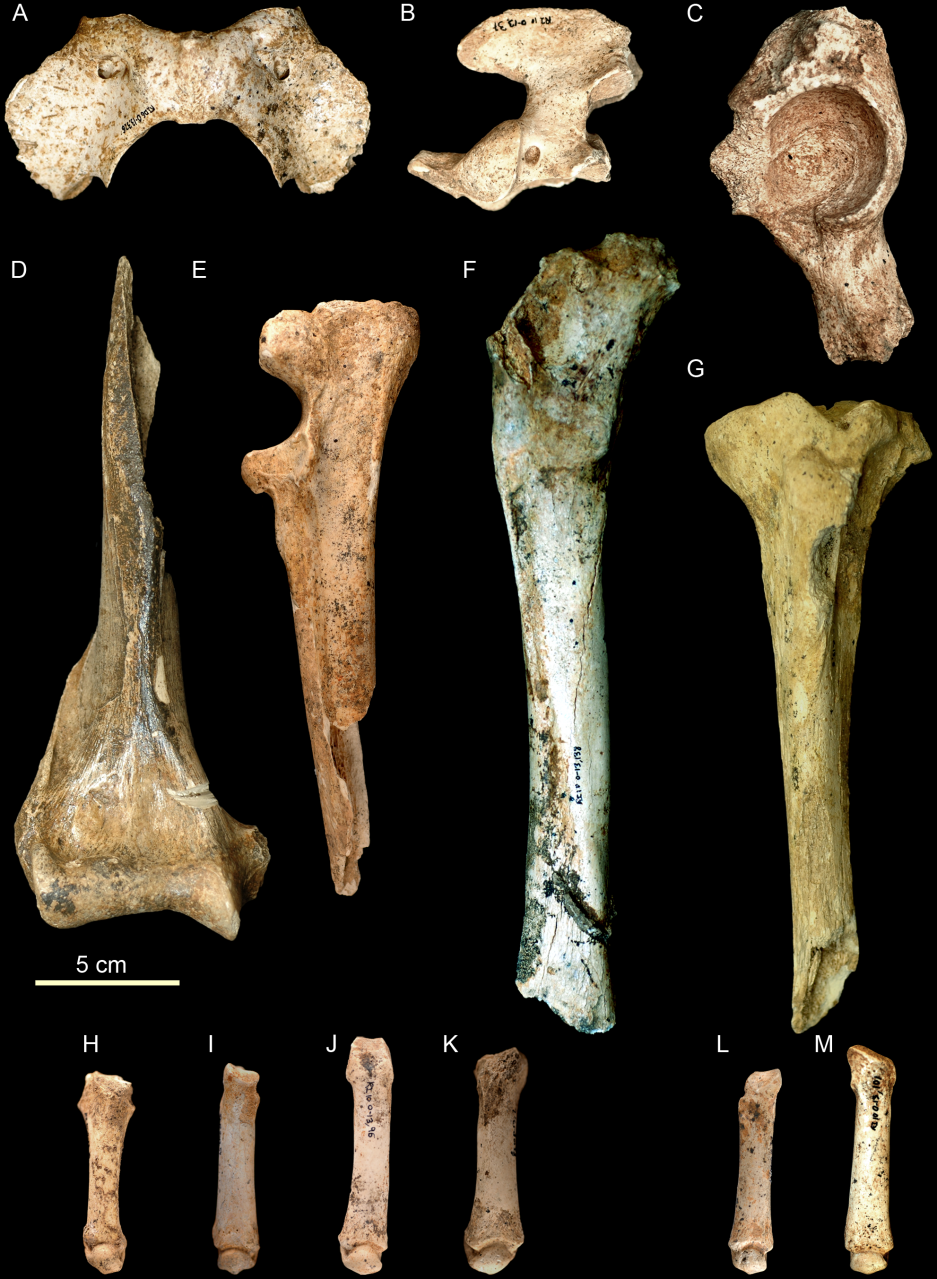
Postcranial remains of *Ursus arctos* from the Late Pleistocene of Los Rincones. Postcranial remains of *Ursus arctos* (adult) from the Late Pleistocene of Los Rincones. A. Atlas Ri10/O13/326. B. Axis Ri10/O13/37. C. Left coxal Ri10/O13/24. D. Right humerus Ri10/O13/328. E. Left proximal part of ulna Ri10/O13/35. F. Right femur Ri10/O13/138. G. Left tibia Ri10/P13/6. H. Left Mc I Ri10/O13/9. I. Right Mc II Ri10/O13/54. J. Right Mc IV Ri10/O13/96. K. Left Mc V Ri10/P13/2. L. Left Mt II Ri10/O14/17. M. Right Mt III Ri10/O13/101.

**Figure 6:**
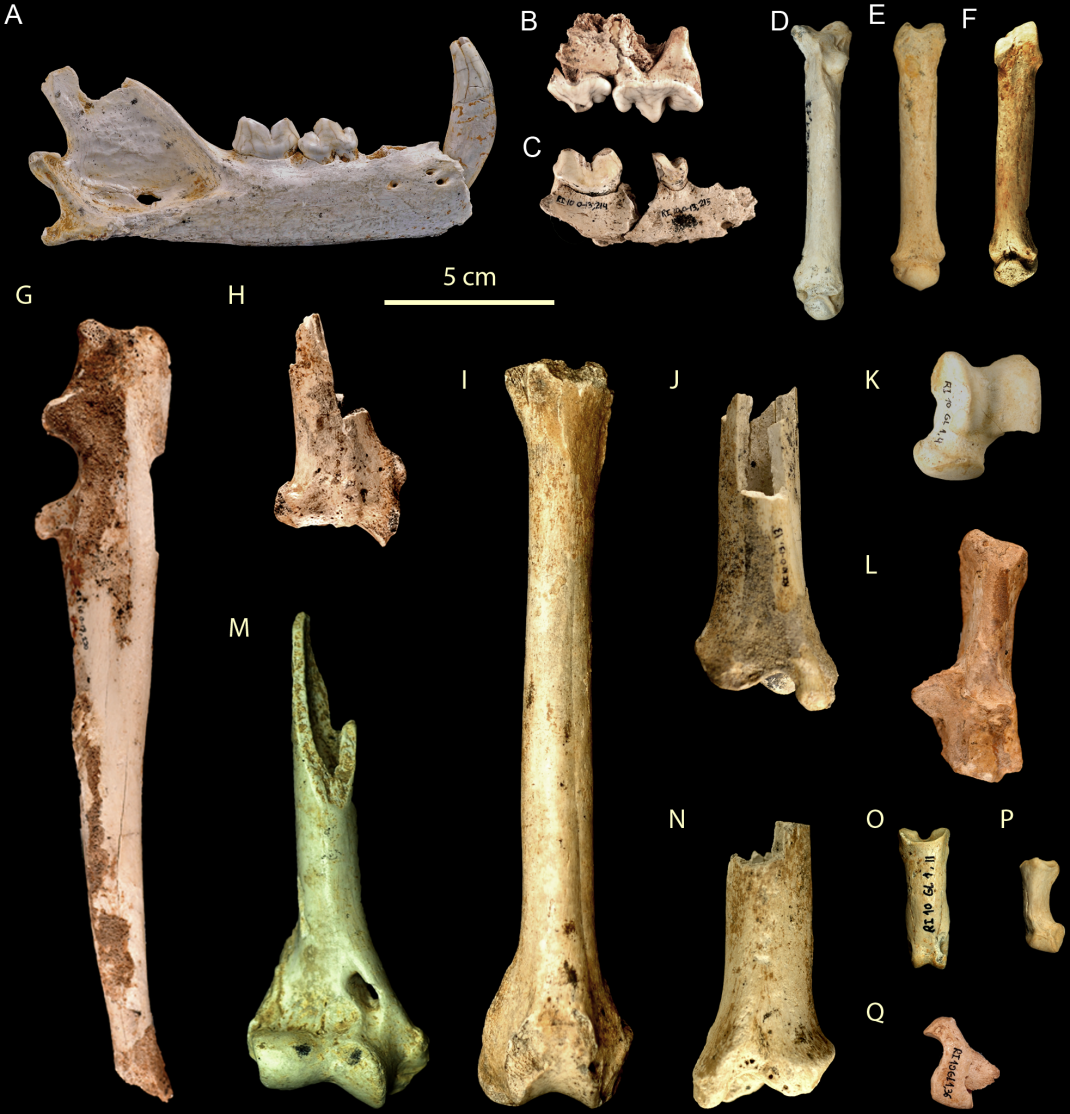
Remains of *Panthera pardus* from the Late Pleistocene of Los Rincones. Remains of *Panthera pardus* (adult) from the Late Pleistocene of Los Rincones. A. Right mandible Ri10/C1/2010. B. Left maxillary with P^3–4^ Ri10/O13/190. C. Right mandible with m1 and p4 Ri10/ O13/214,215. D Right Mt V Ri10/GL1/18. E. Left Mc III Ri10/GL1/16. F. Left Mc IV Ri10/GL1/17. G. Left ulna Ri10/O13/220. H. Right radius Ri10/O14/41. I. Right femur Ri10/O13/223. J. Right tibia Ri10/O13/13. K. Left astragalus Ri10/GL1/4. L. Left calcaneus Ri10/N1032. M. Right humerus Ri10/N10/5. N. Left tibia Ri10/O13/12. O. Phalanx I Ri10/GL1/11. P. Phalanx II Ri10/GL1/38. Q. Phalanx III Ri10/GL1/36.

*U. arctos* (MNI = 8) is the predominant carnivore, represented by 57% of the MNI of the carnivores and 17.39% of the total MNI. This species presents a great variety in the ages of death of individuals, with a neonate individual, three subadults, a juvenile and three adults having been recovered (Fig 4, 5). The next most abundant carnivore is *P. pardus* (MNI = 4), representing 28.5% of the carnivores and 8.69% of the total MNI. All four *P. pardus* individuals are adults (Fig. 6). Other carnivores have also been recovered at the site, including *C. lupus* (NR = 4 and MNI = 1) and *Lynx* sp., from which only two hemimandibles belonging to a single individual have been recovered. The sum of the MNI of the carnivores present at the site represents 30.43% of the total (Table 2).

#### Herbivores

As regards the number of individuals, the species *C. pyrenaica* (MNI = 20) is undoubtedly the predominant taxon in the association, representing 43.48% of the total and 64.5% of all the ungulates from the site. As far as the ages of death are concerned, it shows a broad range, with one neonate, seven subadults, three juveniles, five adults and four senile individuals. The species *R. pyrenaica* is the next most abundant ungulate (MNI = 3), representing 6.5% of all the taxa and 10% of the ungulates; all three individuals are adults. The remaining ungulates are represented by two individuals from each of the species *E. ferus*, *C. elaphus* and *C. capreolus* and by one individual belonging to *E. hydruntinus* and Bos/Bison sp., all of these being adults. The sum of the MNI of the ungulates present at the site amounts to 67.39% of the total number of individuals present in the association.

The sum of the species *C. pyrenaica*, *U. arctos* and *P. pardus* represents 69.56% of the total MNI at the site. Most of these are adults (25) and subadults (10), the sum of which represents 76% of the total individuals in the site. Juvenile and senile individuals are represented by four individuals each. Finally, the most scarcely represented individuals are the neonates, only two of which have been recovered, one belonging to *C. pyrenaica* and the other to *U. arctos* (Table 2). The regression of logA on logBW for the assembly from Los Rincones is insignificant (ρ  =  0.19).

### Skeletal survival rate (%Surv)

As regards the %Surv, this has been calculated separately for *U. arctos* and *P. pardus* in the belief that these taxa might have inhabited the cave, unlike the ungulates, whose presence in the cave may well be the result of the activity of an accumulating agent. To calculate the %Surv for the ungulates, they have been grouped according to size.

**Figure 7:**
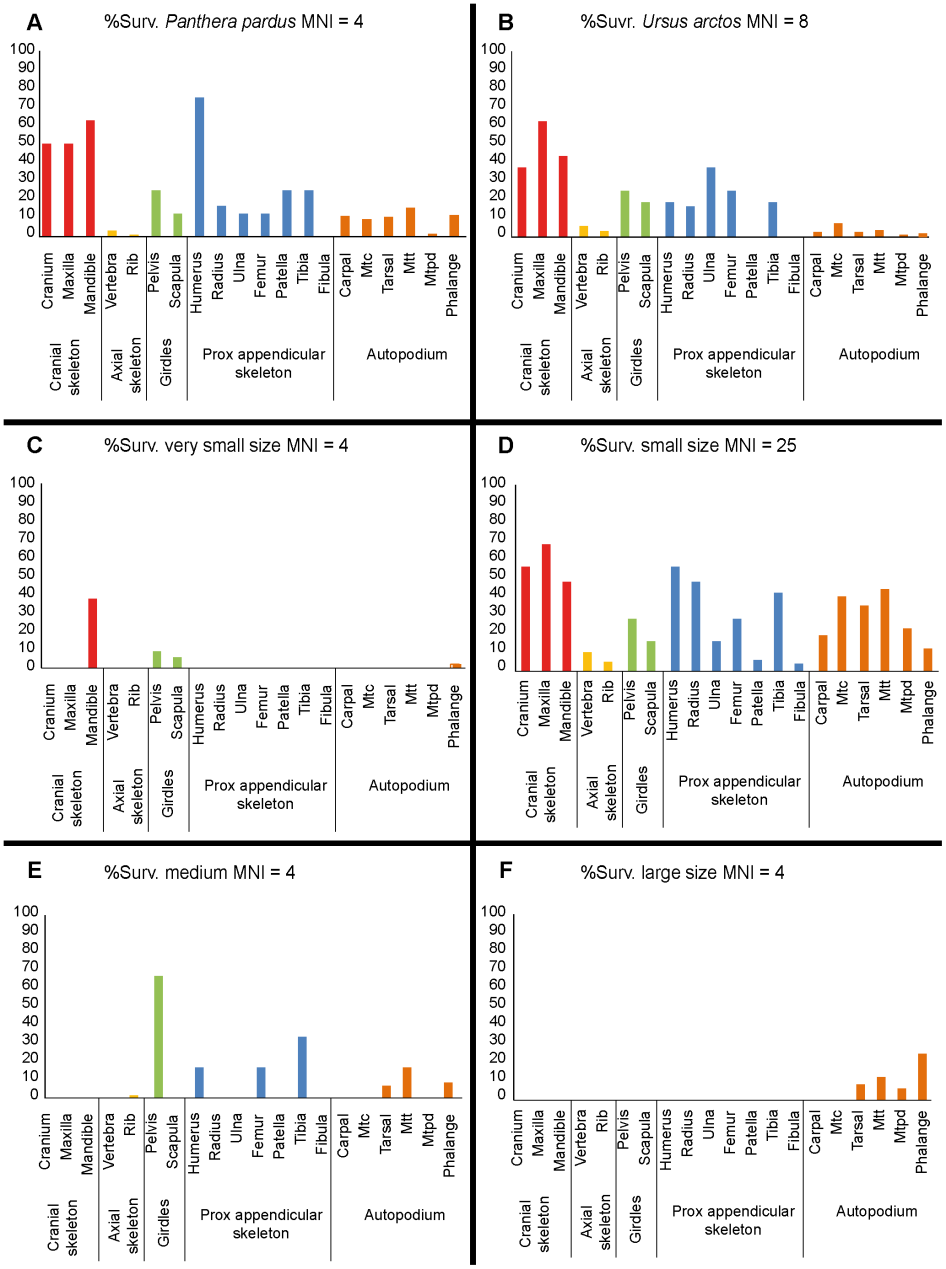
Graphical representation of % Surv. according to skeletal elements recovered in Los Rincones. Graphical representation of skeletal survival rate (% Surv.) according to skeletal elements and size categories established in Los Rincones faunal assemblage.

The %Surv for *U. arctos* shows a predominance of cranial elements; the girdles and long bones (proximal appendicular skeleton) present values close to 20%; while both the axial elements (vertebrae and ribs) and the autopodia show a low representation (Fig.7). The %Surv for *P. pardus* shows a high percentage of cranial elements, although the element with the highest %Surv is the humerus with 75%. The elements of the axial skeleton are present in extremely low percentages (Fig. 7).

As regards the %Surv of the very small-sized ungulates, the element with the greatest %Surv is the mandible with 37.5%, whereas both the appendicular elements and the girdles are missing. Both the elements of the axial skeleton and the autopodia are present in a low proportion (Fig. 7).

The %Surv of the small-sized ungulates shows a reasonably balanced profile in which the low presence of elements of the axial skeleton and the girdles is noteworthy. The elements with the highest %Surv are the maxilla (68%), the cranium (56%), the humerus (56%), the mandible (48%) and the radius (48%) (Fig. 7).

The %Surv of the medium-sized ungulates presents a profile lacking in cranial elements and with a very low frequency (almost zero) of axial elements; the autopodial elements show a low frequency, while the elements with the highest %Surv are the pelvis (66.66%), tibia (33.33%), humerus (16.66%) and femur (16.66%) (Fig. 7).

The %Surv of the large-sized ungulates shows a very imbalanced profile in which only autopodial elements are represented, the phalanges being the element with the highest %Surv (25%) (Fig. 7).

### Breakage patterns

To analyse the breakage pattern of the bones, each of the galleries were taken into consideration: GU at a high zone, GL 1–3 at a low zone, and GL 4–9 at a medium level. The idea was to ascertain whether transport took place between the different levels, which might have been the cause of the variations in the breakage pattern. In GU the breakage angle is predominantly straight (55.14%), the delineation is transverse (50.98%), and the edge is irregular (56.24%). In GL 1–3 there is a predominance of straight breakage angles (52.08%), the delineation is curved (50%), and the edge is irregular (58.33%). In GL 4–9 the breakage angle is predominantly straight (76%), the delineation transverse (58.66%), and the edge smooth (56%). As regards the length of the diaphysis in relation to the breakage of the circumference, major differences can be seen between the three areas. In GU the remains thus present diaphysis lengths and circumference measurements of all types: C1-L1 is the predominant type with 26.41%, followed by C2-L2  = 15.72%, C1-L2  = 13.52%, C3-L4  = 8.49%, C3-L3  = 7.86% and C2-L4  = 7.54%. In GL 4-9 the remains are uniformly distributed in all the types of diaphysis lengths except L4; the most abundant remains are C1-L1  = 39.58%, followed by C3-L2  = 22.91%, C2-L2  = 10.41%, and C3-L3 and C3-L2 with 8.33%. In GL 1–3 the remains show L1 diaphysis lengths with C1 circumferences, 87.03% of the remains in this area being C1-L1. Moreover, it should be borne in mind that eight of the fossils recovered show anthropic breakage, comprising 0.55% of the sample.

### Marks

#### Anthropic cut marks

There are but scarce cut marks.. They can only be seen in 28 remains, representing 1.94% of the sample. The sum of the cut marks and the cases of anthropic breakage is 36 remains, indicating that 2.49% of the remains are affected by anthropic modifications. The cut marks are present mainly on *C. pyrenaica* and small-sized herbivores, although they have also been found on *C. elaphus* and Bos/Bison sp. (Table 3).

**Table 3:** Cutmarks groups according to skeletal element and taxa from Los Rincones faunal assemblage.

Skeletal element	Taxa	NME	Anthropogenic Marks	N° striations	Location	Action performed
Pelvis	*Capra pyrenaica*	1	Incision	1	acetabulo medial	Defleshing
Scapula	small size	1	Choopmarks	9	Scapular neck	Defleshing
Humerus	*C.pyrenaica*	1	Choopmarks	13	cresta epicondiloidea	Defleshing
	small size	1	Incision	1	cresta epicondiloidea	Defleshing
Radius	*Capra pyrenaica*	1	Choopmarks	11	Radius neck	Defleshing
	small size	1	Incision	3	Diaphysis	Defleshing
Femur	small size	3	Incision	11	Diaphysis	Defleshing
Tibia	*Cervus elaphus*	1	Incision	1	Epiphysis distal	Defleshing
Metacarpus	*Capra pyrenaica*	1	Incisions	10	Epiphysis proximal and diaphysis	Defleshing
	*Capra pyrenaica*	1	Incisions	8	Diaphysis	Defleshing
	*Capra pyrenaica*	1	Incisions	3	Epiphysis distal	Defleshing
	*Capra pyrenaica*	1	Incisions	7	Epiphysis proximal and diaphysis	Defleshing
Metatarsus	*Capra pyrenaica*	2	Incisions	7	Diaphysis	Defleshing
	medium size	1	Incisions	2	Diaphysis	Defleshing
Astragalus	Bos/Bison sp.	1	Incision	1	Troclea plantar	Disarticulation
	*Capra pyrenaica*	2	Incision	2	Troclea proximal	Disarticulation
Phalanges	*Capra pyrenaica*	1	Incision	1	Diaphysis	Skinning
	*Capra pyrenaica*	1	Choopmarks/ Scrapes	11	Diaphysis	Skinning
Long bones	medium size	1	Incision	1	Epiphysis proximal	Defleshing
	medium size	1	Incision	2	Diaphysis	Defleshing
	small size	8	Incision	19	Diaphysis	Defleshing
	small size	2	Choopmarks	12	Diaphysis	Defleshing
Unidentified	small size	1	Incisions	2	Diaphysis	Defleshing
	small size	5	Percussion marks	5	Diaphysis	Marrow removal
	small size	2	Percussion notches	2	Diaphysis	Marrow removal
	small size	1	Cortical flake	1	Diaphysis	Marrow removal

#### Carnivore marks

Carnivore marks constitute the main modification of the fossil bones from the site of the cave of Los Rincones. They are present in 16.28% of the remains. All the ungulates except Bos/Bison sp. show alterations produced by carnivores. The European ass, *E. hydruntinus*, is the mammal with the highest percentage of modified remains (50%), although it should be borne in mind that only six elements have been recovered from this taxon. The herbivore with the next highest percentage of remains modified by carnivores is *R. pyrenaica* with 34.5% from a sample of 29 remains. The roe deer, *C. capreolus*, presents 26.9% of its remains modified from a total of 26 remains. The horse, *E. ferus*, presents 22% of its remains modified, but as occurs in the case of the European ass, *E. hydruntinus*, this value should be taken with caution given the low number of specimens. The Spanish wild goat, *C. pyrenaica*, has 17.8% of its remains modified from a sample of 528 (Table 4).

**Table 4:** NR with carnivore damage according to taxa, size and skeletal element.

	*Ursus arctos*	*Panthera pardus*	*Capra pyrenaica*	*Equus ferus*	*Cervus elaphus*	*Rupicapra pyrenaica*	*Capreolus capreolus*	Small size	Unidentified	NR
Skull			2 (2/11)						1	2 (2/72)
Antler/Horns							2 (2/3)			2 (2/21)
Atlas	1 (1/1)		1 (1/4)							2 (2/5)
Axis			2 (2/6)					1 (1/1)		2 (2/9)
Vertebrate	2 (2/10)	1 (1/3)	7 (7/31)						2	12 (12/123)
Sternum		1 (1/3)								1 (1/3)
Sacrum			1 (1/2)							1 (1/2)
Coxal	1 (1/2)									1 (1/2)
Ribs								5 (5/70)		5 (5/82)
Scapulae	3 (3/5)		5 (5/7)						1	8 (8/15)
Pelvis	1 (1/2)		1 (1/11)					2 (2/2)		3 (3/19)
Humerus		1 (1/11)	12 (12/28)		1 (1/1)	2 (2/2)		1 (1/7)		17 (17/56)
Radius	2 (2/6)	1 (1/2)	4 (4/22)				1 (1/1)	7 (7/47)		15 (15/79)
Ulna	4 (4/10)		2 (2/12)					2 (2/2)		8 (8/25)
Femur	5 (5/6)	1 (1/1)	6 (6/15)					4 (4/7)	2	18 (18/42)
Tibia	4 (4/4)		3 (3/29)				1 (1/1)	5 (5/11)	1	14 (14/51)
Carpal/Tarsal			1 (1/40)							1 (1/56)
Astragalus			8 (8/40)			3 (3/4)				11 (11/24)
Calcaneus	1 (1/2)		4 (4/10)			1 (1/1)				5 (5/18)
Metacarpus	1 (1/6)		11 (11/33)							14 (14/41)
Metatarsus	1 (1/3)	1 (1/5)	5 (5/23)				1 (1/5)			7 (7/37)
Metapodials							1 (1/1)	4 (4/31)		2 (2/40)
Phalanges		2 (2/26)	19 (19/64)	2 (2/9)		4 (4/7)				28 (28/132)
Long bones									46	46
NR	26 (26/173)	8 (8/110)	94 (94/528)	2 (2/9)	1 (1/13)	10 (10/29)	7 (7/26)	31 (31/298)	53	235 (232/1034)

NR (Number of remains) with carnivore damage according to taxa, size categories and skeletal elements from the Los Rincones faunal assemblage. In brackets the total number of elements for every taxa. Note several type of alterations can be located on same skeletal element and therefore, NR can be higher than total NR with carnivore damage.

It is also interesting to note the high percentage of modification shown by the remains of *U. arctos,* i.e. 15% from a sample of 173 elements; in lesser measure, the remains of *P. pardus* can also be seen to be modified, with 7.2% from a total of 110 elements (Table 4).

The types of carnivore tooth marks found in the bones at Los Rincones are presented in Table 4. Considering all the taxa as a whole, the most modified elements are the scapulae (53%), followed by the femora (43%), the metacarpals (34%), the ulnae (32%) and the humeri (30%). The cranial and axial elements show less modification, with a percentage equal to or less than 12%. Pits and scores are the most abundant tooth marks, present in 129 and 82 bony remains respectively. Further, the breakage caused by carnivores is recorded by the presence of crenulated edges (NR =  77), scooping out (NR = 31) and impact points (NR = 18). Up to now no remains have been found showing evidence of digestion (Table. 5).

**Table 5:** Carnivore damage according to taxa and size categories from Los Rincones.

	Score	Pit	Puncture	Crenulated edge	Impact point	Furrowing	Scooping out	Scoring	Pitting	NR
*Ursus arctos*	7	11	3	9	2	11	8		4	55
*Canis lupus*										
*Panthera pardus*	2	2	3	2		2	2		1	14
*Lynx* sp.										
*Capra pyrenaica*	27	46	25	17	7	36	13	1	12	184
*Equus ferus*	2	2	1			4		1	2	12
*Cervus elaphus*		1	2							3
*Rupicapra pyrenaica*	1	4	5		1	6			1	18
*Capreolus capreolus*	3	5		1			2		2	13
Bos/Bison sp.										
Very small size										
Small size	14	20	6	2	3	8	6		5	64
Middle size										
Large size										
Unidentified	26	38		14	4	3			6	91
*Total*	82	129	45	45	17	70	31	2	33	454

## Discussion

### Paleoenvironmental context

The herbivores present at Los Rincones are associated with various types of landscapes. The horses (*E. ferus* and *E. hydruntinus*) and large bovids such as Bos/Bison sp. [71] indicate open environments [68], [72], while *E. hydruntinus* also suggests semi-arid conditions [73]. On the other hand, *C. elaphus* and *C. capreolus* indicate a wooded habitat [74], [75], [76], [77], [78]. However, the best-represented herbivores both in terms of NR and MNI are those associated with areas of high or medium mountains with abrupt relief, such as *C. pyrenaica* and *R. pyrenaica* [79], [80], [81].

The carnivores of the cave of Los Rincones, *P. pardus* and *C. lupus*, prefer a broad range of habitats [1], [2], [3], [4], [82], [83]. The only small-sized carnivore present is the *Lynx* sp., an opportunistic carnivore that populates wooded habitats ranging from Mediterranean to high mountainous areas [84], [85].

During the Pleistocene, the brown bear, *U. arctos,* populated a broad variety of habitats, ranging from tundra to woodland of all types, both in valleys and in areas of medium-high mountains; the Iberian Peninsula was a southern European refugium during the glaciations [86], [87], when refuge was found in caves and cracks of all kinds [88], [89].

### Breakage patterns

The sample presents a degree of fragmentation of 68.5%, with 279 complete remains. The faunal composition, the skeletal survival profiles and the degree of preservation of the remains, as well as the distribution of fragments of the same anatomical element in distinct galleries of the site, indicate that the process of accumulation was similar: bones, with other clastic sediments, were carried in from the surface (allogenic transport) to the GU, until the cone blocked the mouth of the cave. To study the fragmentation of the bones, we divided the site into two galleries: GU was where most of the material was recovered and where the remains are found in the position they occupied in the period prior to the closure of the cave; the remains that were on the surface of the sediment accumulated among the blocks in GU have moved towards lower levels (GL), passing between the gaps left by the fallen blocks, causing greater fragmentation and resulting in a reduction in the length and circumference of the remains. The breakage data were compared with those from Neolithic sites: Fontbrégoua, where the breakage is anthropic in origin; Sarrians, where the breakage was caused by the weight of the sediment load; and Besouze, where the breakage was produced by the impact of falling blocks [63]. Comparisons were also drawn with other sites of a similar chronology such as Pinilla del Valle [90], [57], the Búho and Zarzamora caves [57], [91] and Coro Tracito [62], the first three interpreted as carnivore dens, possibly hyena dens, and the fourth a cave inhabited by cave bears (*U. spelaeus*), where the breakage was caused by a combination of the activity of the bears and the pressure of the sediment [62]. Further comparisons were made with sites of similar chronology but where the cause of breakage was anthropic, such as Abric Romaní level B and Vanguard Cave [92]. Moreover, comparisons were drawn with Middle Pleistocene sites with breakage of an anthropic origin such as levels TG10C-D-TN5 of the site of Galería and Gran Dolina level 6, both located at Atapuerca, Burgos [36], [92], as well as with others where the breakage has been attributed to the activity of carnivores, such as Gran Dolina level 8 [72].

The sites where the breakage occurred on fresh bone (green bone) present fractures with mainly oblique angles, smooth edges and curved delineations. The main agents of breakage are the primary consumers, i.e. the humans that extract the marrow or the carnivores that gnaw on and partially consume the bones, such as the hyena and wolf [36], [63], [90], [92].

**Figure 8:**
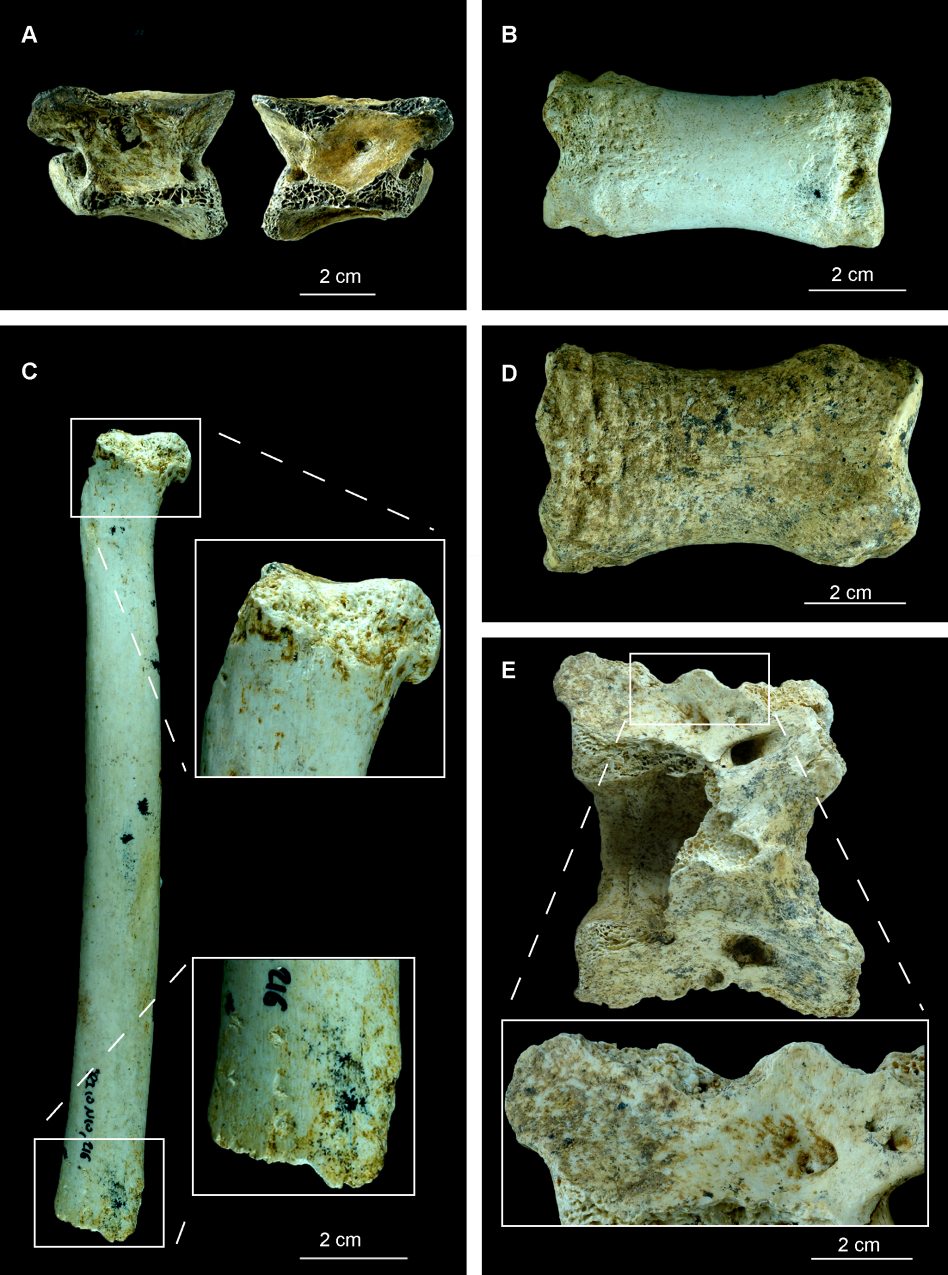
Examples of carnivore damage from Los Rincones faunal assemblage. Examples of carnivore damage from Los Rincones faunal assemblage : A, vertebra of *C. pyrenaica* with puncture in both sides of the vertebral body Ri10/N1/39; B, phalanx of *E. ferus* with furrowing and pits Ri10/N10/68; C, radius of *P. pardus* with pits and scores in both ephysis Ri10/N10/216; D, phalanx of *E. ferus* with scores and pits Ri10/O13/71; E, atlas of *C. pyrenaica* with crenulated edges Ri10/O13/82.

**Figure 9:**
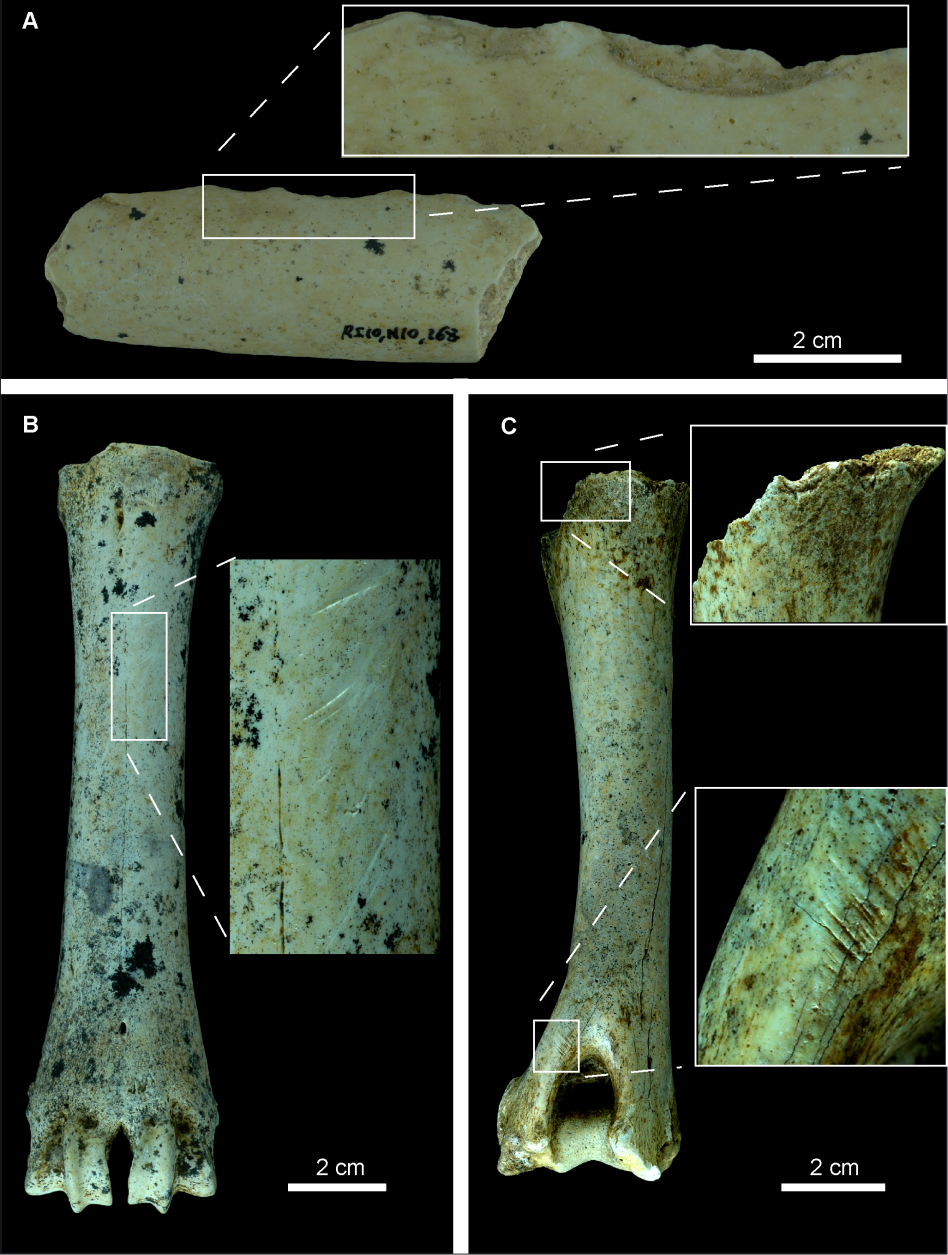
Examples of anthropogenic damage from Los Rincones assemblage. Examples of anthropogenic damage from Los Rincones assemblage. A, percussion marks related to marrow removal Ri10/N10/168. B, metacarpus of *C. pyrenaica* with oblique incisions related to defleshing Ri10/N10/195. C, humerus of *C. pyrenaica* with oblique chopmarks related to defleshing and also carnivore marks (scooping out) in proximal epiphysis Ri10/O13/179.

However, the analysis of the bony remains from the cave of Los Rincones yielded results closer to those sites where the breakage occurred when the bone was no longer fresh, with a predominance of fractures with straight angles, transverse delineation and irregular edges. The values from Los Rincones are most similar to those from the site of Besouze, which was interpreted by Villa & Mahieu [63] as a site where the breakage had been caused by falling blocks. Yet even though falling blocks were the main cause of the bone breakage at Los Rincones, breakage of fresh bone is also in evidence; this is both anthropic in origin, giving rise to impact points, and produced by carnivores, resulting in crenulated edges (Table 3, Table 4, Fig.8, Fig.9).

### Skeletal survival rate (%Surv)

The accumulation at Los Rincones is made up mainly of *C. pyrenaica* and *U. arctos*. The brown bear is represented by all its skeletal elements, which indicates that it occupied the cave as a hibernation refuge [93].The small-sized ungulates present a reasonably balanced skeletal survival profile, especially when compared with the medium and large-sized taxa, which show a bias towards the appendicular elements.The skeletal elements present in the accumulation at Los Rincones do not correspond with those present in an accumulation that is geological in origin, since phenomena resulting in differential preservation, such as transportation in a watery medium, are directly related to the density of the bones [34], [94], [95], [96].

Anatomically and taxonomically, the accumulation of bony remains of herbivores suggests an accumulating agent. It should be pointed out that this selection does not show an age bias, and individuals of all ages are found.

### Anthropic cut marks

The human presence in the cave is also in evidence, for the type of cut marks and their location indicate that some of the herbivores were exploited for their meat, showing evidence of skinning, carving, dismembering and defleshing operations. Furthermore, there are also signs of bone breakage for marrow extraction. However, the cut marks and signs of anthropic breakage are only found in 2.26% and 2.91% of the total sample. The scarcity of anthropic alterations, the presence of just a single piece of lithic industry, and the absence of evidence of a human habitat at the site make it highly unlikely that the accumulation was produced by a population of hunter-gatherers. The cave may thus have been occupied intermittently as a place of hunting or slaughter, or a more likely possibility is that the faunal remains that display anthropic marks were scavenged by carnivores after being discarded by prehistoric humans. Moreover, the presence of marks of anthropic activity at sites interpreted as carnivore dens has been documentedfor instance Buena Pinta Cave [97], Zarzamora Cave [91], Amalda VII [25], [26] and Cova de Dalt del Tossal de la Font [98] in the Iberian Peninsula; Les Auzières 2 and Bois Roche in France [99], [100], [101]; the Geula Cave in Israel [102]; and Zourah Cave in Morocco [103].

However, the presence of 22.94% of anatomical elements modified by carnivores as well as of their direct remains can be taken to indicate that the cave served as a refuge and a place of storage for carnivore kill.

### Carnivores during Pleistocene in the Iberian Peninsula

The various species of carnivores may be responsible for the accumulation both of herbivore remains and the remains of other carnivores. To gain insights into the role of carnivores as accumulating agents of other mammals, the characteristics of the accumulation are studied on different scales: on the one hand, the skeletal elements and the characteristics and severity of the bone damage, as well as the measurements of the tooth marks; and on the other hand, the taxonomic composition and the age of death of the individuals that make up the taphocoenosis e.g [50], [64], [104], [105]. In addition, attention must be paid to the ethological characteristics of the carnivores in question, in particular their potential as bone accumulators and the range of prey they usually consume e.g. [60], [61], [67], [69], [83], [106], [107].

Taking account of the characteristics of the carnivores that inhabited the Iberian Peninsula during the Late Pleistocene, we here discuss the possible causes of the taphocoenosis of galleries GU and GL of the cave of Los Rincones. The present-day brown bear has been present in the Iberian Peninsula since the Middle Pleistocene. With a first citation as *Ursus* cf. *arctos* at the site of Gran Dolina 11 at Atapuerca [109], it is also found at the Middle/Late Pleistocene sites (MIS 11 to MIS 5) of Cueva del Ángel [110] and Valdegoba [111]. During the Late Pleistocene, it shows a broad distribution, occupying practically the whole of the Iberian Peninsula [86], [87], [112]. It is an animal that uses caves as a refuge during its hibernation period. During this period and especially at the end, mortality is very high, as a result of which the dead bodies remain inside the caves [113], [114], [115], [116]. Bears are fundamentally omnivorous, with a diet based on plants, insects and small mammals, generally carrion, but on rare occasions a result of direct predation [88], [89], [117]. Even though bears can consume small and medium-sized mammals, such as those found at Los Rincones, when they consume meat, they do so without transporting remains from the carcass and thus without making any contribution to their hibernation dens [60], [69], [107], [118], [119]. Even if we rule out the bear as the main accumulator of the remains, it is possible that it modified the carcasses that other predators might have accumulated in the cave. The spotted hyena *Crocuta crocuta* was recorded in the Iberian Peninsula from the Early Pleistocene [109], and the taxon is present in many Late Pleistocene sites in the Iberian Peninsula. The most recent record of the taxon in the Iberian Peninsula is from Las Ventanas Cave, dated to 12.5 ka [120]. The spotted hyena is a social carnivore that is organized in clans that can be very numerous (comprising up to 80 individuals) and display territorial behaviour [121]. Hyenas are both scavengers and hunters, and can feed on almost all resources available to them, ranging from insects, all sorts of ungulates, to carnivores and even elephants [2], [66], [122], [123], [124], although recent studies indicate that 95% of the prey consumed are the result of direct hunting [125], with a preference for prey between 56 and 182kg [126]. Hyenas can consume their kill “in situ”, yet they generally transport it to their dens in outlying areas in order to feed their young. Bone accumulations are thus formed in these places [67], and many authors have noted the presence of bony remains in hyena dens, [8], [121], [127]. The oldest reference to *P. pardus* in the Iberian Peninsula is from the Middle Pleistocene of level VI of Lezetxiki, dating to 234 ±32 ka [128], [129], but it was not until the second half of the Late Pleistocene that this species extended its range throughout the Iberian Peninsula [19], [129], [130], [131], finding a refugium on the Cantabrian coast until its disappearance from Europe at the end of the Late Pleistocene [19]. The leopard is a solitary and territorial hunter [132], [133] with an exceedingly broad range of prey comprising as many as 92 species in Sub-Saharan Africa and with only exceptional cases of cannibalism on record [134], although they focus mainly on prey ranging from 20-80kg in weight [108]. Though not a selective hunter [133], the leopard shows a preference for prey with an optimal weight of 23kg [133]. In open spaces, leopards protect their kill by hauling them up into trees [6,7,8,910], but in areas where there are caves they prefer to accumulate their carcasses inside these see references in de Ruiter & Berger [11].

The presence of *C. lupus* is recorded from the Middle Pleistocene in localities such as level TG10a of the Trinchera Galería at Atapuerca [135] through to the present day. Its diet covers a very broad spectrum, and it can consume ungulates, lagomorphs, carnivores, reptiles and birds see references in Esteban-Nadal [136], although it shows a preference for large ungulates [137], [138], [139]. Wolves consume their prey “in situ”, and only occasionally transport their kill to their dens, when rearing their young. Remains tend to comprise fragments of regurgitated bones, which do not generally form large accumulations [107]. At present, wolves cannot be considered producers of the taphocoenosis of Los Rincones, as there are no major bony accumulations known to have been caused by this carnivore [107], [140], [141], although they are possible taphonomic agents, since they are capable of modifying the samples [20], [21], [141], [142], [143].

The Iberian lynx (*Lynx pardinus*) has been found in the Iberian Peninsula since the late Early Pleistocene (ca 1.0 Ma) [144], [145]. During cold periods of the Late Pleistocene the presence of the Eurasian lynx (*Lynx lynx*) has also been established [146], [147], though only in the region of Cantabria. The Iberian lynx is a specialist hunter whose most common prey is the rabbit (*Oryctolagus cuniculus*), which represents 85–100% of its diet [84], [85], complemented with birds, reptiles and small mammals of less than 50kg [84], [148]. Accordingly, it could be responsible for the accumulation of roe deer, chamois and the females and juvenile individuals of *C. pyrenaica* at the cave of Los Rincones. Having reviewed the taphocoenosis present at Los Rincones, composed mainly of small-sized ungulates, and taking into account the ecological and ethological characteristics of the carnivores that inhabited the Iberian Peninsula during the Late Pleistocene, we propose two carnivores as the presumed producers of the bony accumulation in question: the spotted hyena and the leopard. It should also be borne in mind that the remains could have subsequently been modified by other carnivores such as the wolf, the lynx and even the bear.

Below we discuss which of the two main candidates was responsible for the accumulation: the hyena or the leopard. To this end, we compare the types of damage present on the bone surface, the amount and position of this damage, the size of the marks, and the skeletal survival profiles of the prey, and these are compared with the bibliographical data on the accumulations produced by these two predators [8], [15], [64], [67], [107], [148].

### Comparison with other sites from European Pleistocene

In most of the Late Pleistocene dens ascribed to the hyena, hyena bones are abundant, it being the most highly represented carnivore in most cases [149], [150], [151], [152], [153], [154], [155]. This has been verified at various European sites (n = 22), where hyenas represent 33.2% of the total NR [146]. The presence of deciduous hyena teeth is a good indicator that the cave was used as a den [65], [66], [90], [101]. Another criterion that is generally a good indicator of hyena activity in or near the cave is the presence of coprolites [65], [101], [122], [123] since the hyena uses faecal pellets to mark their territories and dens [124], [153]; the presence of coprolites is common at Late Pleistocene sites such as La Valiña [156], Caldeirao [157], Cueva del Camino [90], Gabasa 1 [158], Las Ventanas [120], Labeko Koba level IX [159], Nerja [160], Zarzamora Cave [91], Bois Roche [101], Sloup Cave, Sipka Cave, Sveduv Stůl Cave [153] and Westeregeln [154]. One characteristic of the accumulations produced by hyenas is the presence of digested bones [65], [101]. These have been preserved at various Pleistocene sites such as Cueva del Camino [90], Buena Pinta Cave [97], Zarzamora Cave, where they amount to as much as 20.54% of the total NR [56], the Mousterian levels of Caldeirao [157], Gabasa 1 [21] and the Terrasses de la Riera dels Canyars [161]. Although the presence of coprolites and digested bones are good indicators of hyena activity, their absence does not rule out the presence of hyenas [15]: no coprolites have been recovered from the site of Auzières 2 [100] neither from Teufelskammer Cave [150], which are associated with hyena activity, nor have they been recovered from any of the dens of present-day hyenas [15]; the same applies to digested bones [162]. Comparing Los Rincones NISP pie diagram (Fig. 3) with pie diagram of hyena den such as Wilhelms cave, Hohle Stein cave, Teufelskammer cave [150], Westereleng [154] and Sloup cave [153] the main difference is that in Los Rincones the precentage of leopard is higher than in the other places and no bony remains of hyena have been recovered. Otherwise, the hyena taxa is always present in hyena den [150], [153], [154]. Regarding hervibores of Los Rincones, *C. pyrenaica* is the most abundant taxa and is the main prey of leopards [24]; commonly in the hyena den Pleistocene hervibores have medium of big size like *Coelodonta antiquitatis*, *Rafinger tarandus*, *Mammuthus primigenius* [150], [153], [154]; also nor have coprolites been recovered in the Los Rincones, or herbivore bones with signs of digestion.

Furthermore, the skeletal profiles left by hyenas are highly biased in favour of appendicular and cranial elements due to the transport of these anatomical elements to the dens [8], [25], [67], [163].

By contrast, when the leopard creates accumulations by bringing its prey to a shelter or nearby cave, it generally transports whole carcasses. Accordingly, the skeletal profiles it produces are more balanced than those left by hyenas [11], [15], [25], [26], [67]. The small herbivores at Los Rincones represent more than 80% of the total and present balanced skeletal profiles, in accord with the accumulations generated by leopards. Large herbivores such as *E. ferus*, *E. hydruntinus* and Bos/Bison sp. present skeletal profiles consisting exclusively of autopodial elements. This pattern does not correspond to the accumulations produced by leopards since these animals fall outside the range of prey captured by leopards, which lies between 20–80 kg [108]. The remains of equids lack cut marks, and 33% of them present tooth marks mainly from gnawing, with a high frequency of marks per modified fossil element. This alteration pattern is very different from what is produced by leopards, since these do not modify the phalanges and tend not to produce signs of gnawing or a high frequency of marks [67], [15]. Accordingly, the accumulation of these large-sized ungulates in the cave must have been produced by another type of predator. The only individual of Bos/Bison sp. displays cut marks, and its accumulation in the cave may be associated with the sporadic use of the cave by a group of humans.

### Mortality profiles

The mortality profiles generated by leopards and hyenas are different on account of their different hunting strategies. Cursorial carnivores such as hyenas generate attritional mortality profiles in which there is an abundance of potentially weak individuals such as the young and the senile and ill [65], [123], [164], [165].

By contrast, predators with a hunting technique based on ambush, such as the leopard, produce mortality profiles that reflect a smaller selection of prey. These are characterized by a representation of ages similar to a living population, generally comprising a high number of juveniles, a low frequency of prime adults and relatively few old adults e.g [7], [166]. In the case of Los Rincones, the mortality profile does not show a bias towards juvenile and senile individuals, suggesting that the predator that accumulated the remains used an ambush hunting strategy such as that used by leopards.

### Tooth marks

The accumulations generated by hyenas tend to show abundant tooth marks [150], [151], [152], [153], present in 60–100% of the sample [25], [67], [163], [167], [168]. In fossil sites attributed to hyena activity, the percentage of tooth marks is generally greater than 40%. Examples include El Esquilleu Cave level III (53%) and unit IV (40%) [25] and Zarzamora Cave (42%) [56]; Cueva del Camino presents carnivore-produced modification of 56% [90], [97]; Buena Pinta Cave shows modification of 53% [97]; in level 2 of Bois Roche 72.3% of the bones are modified by tooth marks, and in level 1c the figure is 66.55% [101].

By contrast, the accumulations produced by leopards tend not to present tooth marks in more than 25% of the sample [8], [67], with the long bones presenting tooth marks in less than 50% of the MNE. Moreover, the number of individual marks in each bone varies greatly according to the accumulating agent: leopards rarely (<5%) leave more than 10 marks on a bone, whereas in the case of hyenas it is common for bones to show many individual marks, with as many as 42 in a single bone [15], [169]. The total sample from Los Rincones shows modification in 22.94% of the NISP. Further, none of the long bones shows marks more frequently than 50%; the femora show the highest percentage of modification with 43%, while the rest of the long bones show values less than 32%. Bones with more than 10 marks represent 3.59% of the sample, and these remains are mainly metapodials (66%), which are not usually modified by felines [15], [67]. Accordingly, we believe that these remains were accumulated by felines and subsequently modified by other carnivores that scavenged on them. Humeri show a different consumption pattern between hyenas and leopards, since felids only produce furrowing in the caudal part of the medial condyle, whereas hyenas produce furrows from the lateral part to the trochlea through the lateral condyle [15]. Therefore, when damage is found in the lateral condyle or both condyles, it is more common for it to be caused by hyena gnawing [15]. In the case of Los Rincones, the distal epiphyses of the humeri are intact, so only the leopard can have contributed to the accumulation.

Accumulations produced by leopards show a high proportion of complete long bones (close to 90%), though these may be modified by post-depositional processes [15]. This is the case for Los Rincones, where the percentage is much lower due to the high level of breakage caused by the above-mentioned transportation among fallen blocks. This breakage occurred when the bone was in a dry state, so we can rule out that it was anthropic or produced by carnivores. Accordingly, this value should not be used to rule out the leopard as the accumulating agent.

To sum up the pattern of bone modification and the number of the marks per bone led us to exclude the role of the hyena in the accumulation.

### Analysis of the measurements of the carnivore marks

**Figure 10:**
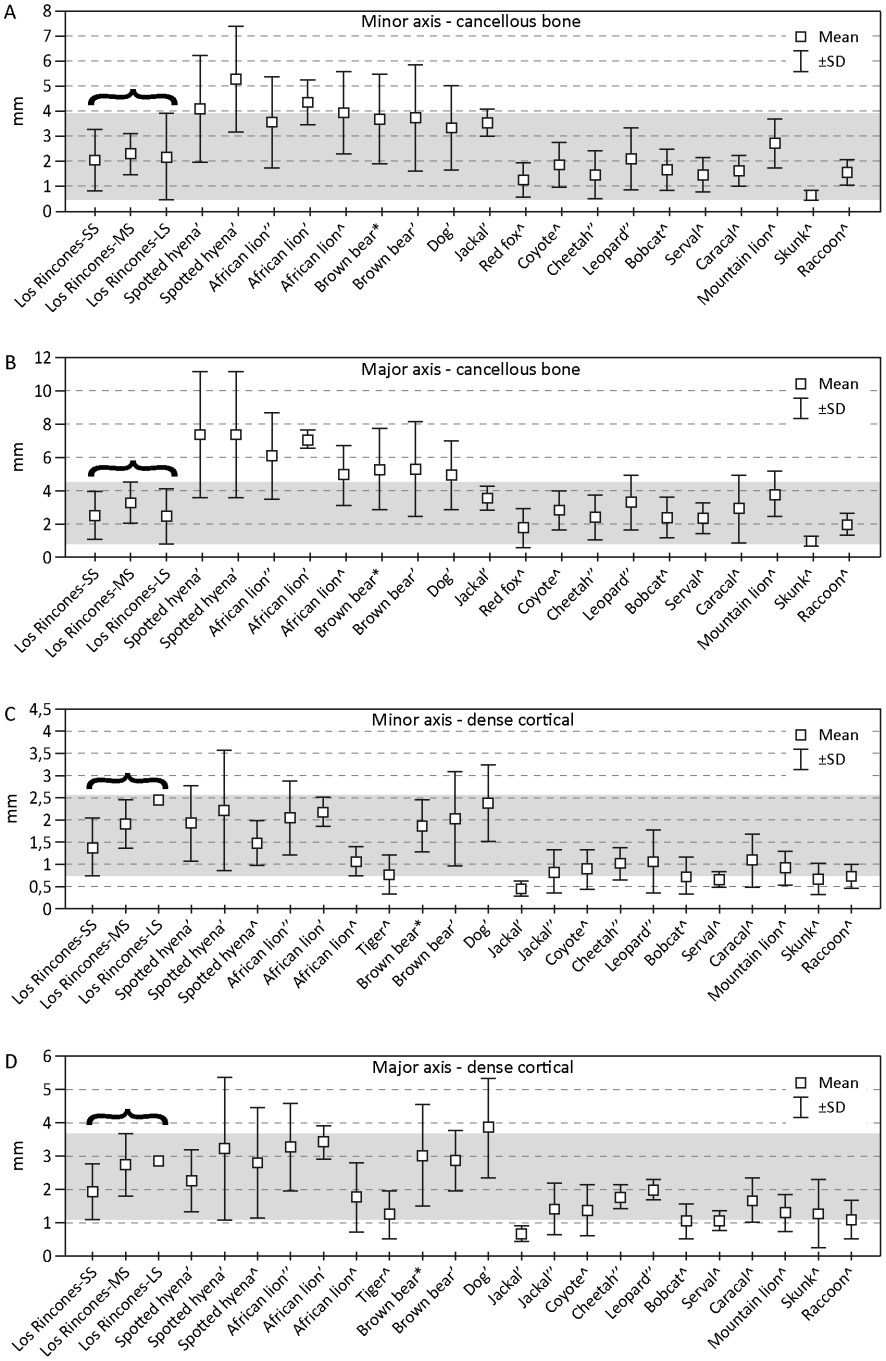
Mean of carnivore tooth pit sizes according bone type and length/breadth from Los Rincones. Mean of carnivore tooth pit sizes according to bone type (cancellous and dense cortical) and length/width from Los Rincones. Legend data from: Delaney-Rivera et al., [57], Domínguez-Rodrigo and Piqueras [58], Saladié et al., [59] and Selvaggio and Wilder [161].

As regards the size of the tooth marks, it should be borne in mind that most of the marks are found on small herbivores n = 362 (thick cortical bone n = 154, fine cortical n = 208), whereas for medium-sized herbivores the figure is n = 48 (fine cortical bone n = 29, thick cortical bone n = 19), and for large herbivores it is only n = 13 (fine cortical bone n = 12, thick cortical bone n = 1). This suggests that the values obtained from the small-sized ungulates show greatest consistency from a statistical point of view. Taking into consideration the data obtained from the experiments by [58], [59], [170], [171] (Table.6, Fig. 10), the dimensions of the depressions found at Los Rincones are compared with those produced by present-day carnivores. Most of the length measurements for the depressions in spongy tissue in the small ungulates are between 1–3 mm, which is the ±SD (Standard Deviation) ; these values are similar to those shown by Iberian lynx, red fox, gray wolf, brown bear and leopard, and the mean of the measurements is almost the same as that presented by the leopard. By contrast, these values hardly overlap at all with those presented by hyenas and lions. Most of the length measurements for the marks in thick cortical bone in the small ungulates fall between the values of 1–2.8 mm, which is the ±SD; these values lie within an area where practically all carnivores overlap.

**Table 6:** Measurements of pits and punctures from Los Rincones faunal assemblage.

	Pits						Surcos			
	cancellous			dense cortical			cancellous		dense cortical	
	Width	Length	L/W	Width	Length	L/W	Width	Length	Width	Length
Small size										
Mean	2.02	2.52	1.31	1.38	1.94	1.46	1.17	5.14	0.89	4.38
SD	1.21	1.42	0.29	0.65	0.84	0.41	0.59	2.92	0.39	1.93
Min.	0.4	0.51	1.00	0.50	0.75	1.00	0.41	1.29	0.27	1.17
Max.	8.35	9.02	2.64	4.64	5.41	2.70	2.90	14.82	2.30	12.50
n	207	202	202	153	153	153	59	59	77	77
Medium size										
Mean	2.28	3.27	1.47	1.91	2.74	1.45	1.67	5.92	1.34	6.31
SD	0.82	1.24	0.39	0.55	0.94	0.33	1.15	3.97	0.89	2.54
Min.	0.85	1.16	1.04	0.85	1	1.00	0.57	2.06	0.21	3.28
Max.	4	5.88	2.49	2.81	5.3	2.19	4.17	12.23	3.45	10.82
n	28	27	27	21	21	21	9	9	17	17
Large size										
Mean	2.18	2.46	1.25	2.44	2.88	1.18	1.71	9.06	-	-
SD	1.73	1.67	0.29	-	-		0.73	1.70	-	-
Min.	0.66	1.18	1.04	2.44	2.88	1.18	0.72	6.56	-	-
Max.	5.47	5.7	1.79	2.44	2.88	1.18	2.96	11.66	-	-
n	6	6	6	1	1	1	7	7	-	-

Measurements of pits and punctures from Los Rincones faunal assemblage according to bone type (cancellous and dense cortical) and length/width.

The width of the depressions in spongy tissue and fine cortical bone, which is in general between 0.7 and 3 mm, falls within the area of overlap for most carnivores (Iberian lynx, red fox, leopard, hyena, gray wolf and lion). Nonetheless, the mean and the size range are related above all with medium-sized carnivores such as the puma, the Iberian lynx and the leopard.

By contrast, the size of most of the depressions in spongy tissue and fine cortical bone in the medium-sized individuals is between 0.5 and 4 mm, and in thick cortical bone between 1.3 and 2.5 mm. Furthermore, they show mean values greater than those of the marks present in the small individuals. These values – both for the width and the length – lie within the area of overlap for most carnivores (hyena, lion, gray wolf, brown bear, leopard and lynx).

The sizes of the depressions found in the small-sized ungulates relate most of the marks with a medium-sized feline such as the leopard, whereas in the medium- and large-sized ungulates the size is greater and could be produced by a gray wolf, hyena, brown bear or leopard. However, they could also be produced by a mixture of other medium- and large-sized carnivores. Given the species of carnivorous mammals that appear at the site, the producers of the bites are likely to be leopards, brown bears or wolves.

## Conclusions

The cave of Los Rincones is a cavern that was closed off by a detritus cone during the Late Pleistocene. The fossil remains under study present anthropic modifications as well as modifications produced by carnivores. The scarce presence of remains of lithic industry, together with the scarce anthropic modification, the high level of modification caused by carnivores and the high percentage presence of carnivores, leads us to believe that the accumulation at Los Rincones was generated by carnivores mainly leopard and that the human presence was very sporadic.

The fossil material recovered from the cave of Los Rincones was found mostly on the surfaces of the *Ursus* Gallery and the Leopard Gallery. Access to the galleries where the fossil remains were recovered is via an entrance distinct from the original one, which was blocked by the sedimentary cone directly at the cave mouth [19]. Before these galleries were sealed off, they could have been used as a refuge by animals such as the present-day brown bear *U. arctos*, which has cave-dwelling habits during its hibernation, and the leopard *P. pardus*, which could have used it to protect its kill from other predators such as the hyena *C. crocuta*. The faunal association recovered shows a high diversity of taxa, though most of the remains belong to small- to medium-sized ungulates, in particular *C. pyrenaica*; the best-represented carnivore is *U. arctos*, followed by *P. pardus*. With this predominance of *C. pyrenaica* and *U. arctos*, this association indicates a medium-high mountain environment at the time when the remains were being accumulated.

The ungulates recovered from the cave were transported there by carnivores, which used the cave as a refuge for protecting their kills.

The small-sized ungulates present reasonably balanced skeletal profiles, catastrophic mortality profiles with no predominance of juvenile or senile individuals, a moderate percentage of marks between 17% and 34%, the type of marks predominantly consisting of pits and furrows, the number of marks present per element rarely exceeding 10, and the size of the marks in accord with those produced by a medium-sized feline. All this would suggest that the accumulation of small ungulates was produced by leopards, which feed mainly on this sort of prey.

The large-sized ungulates present a highly biased skeletal profile, consisting only of autopodial elements; moreover, these elements show a high percentage of modification. These ungulates are not among the range of prey of leopards, and the abundance of autopodial elements and the type of modification they have undergone leads us to believe that they were accumulated by the activity of carnivores other than leopards.

We can conclude that leopards and not hyenas had contributed to the accumulation, subsequent modification of the remains by other carnivores is not ruled out; furthermore, the impact of falling blocks and the transportation of the bones have notably modified their surface and increased the degree of fragmentation of the sample.

## References

[1] Kingdon J (1977) East African Mammals. An Atlas of Evolution in Africa, Vol. III Part A (Carnivores), London, Academic Press. 762 p.

[2] Myers N (1986) Conservation of Africa’s cats: problems and opportunities. In Cats of the world: Miller, SD, Everett DD (Eds). Washington, DC: National Wildlife federation. pp. 437–457.

[3] Nowell K, Jackson P (1996) Wild Cats: Status Survey and Conservation Action Plan.IUCN (World Conservation Union), Gland, Switzerland. 385 p.

[4] Turner A, Antón A (1997) The big cats and their fossil relatives. Columbia University Press, New York. 234 p.

[5] Pocock RI (1932) The leopards of Africa. J.Zool: 543–591.

[6] Pienaar UV (1969) Predator-prey relationships amongst the larger mammals of the Kruger National Park. Koedoe 12: 108–176.

[7] Schaller GB (1972) The Serengeti Lion: A Study in Predator–Prey Relations, Chicago: University of Chicago Press. 504 p.

[8] Brain CK (1981) The Hunters or the Hunted? An Introduction to African Cave Taphonomy. University of Chicago Press, Chicago. 356 p.

[9] Le Roux PG, Skinner JD (1989) A note on the ecology of the leopard (*Panthera pardus* Linneaus) in the Londolozi Game Reserve, South Africa.Afr J Ecol. 27: 167–171.

[10] Mills MGL (1990) Kalahari Hyaenas: The Comparative Ecology of Two Species. London: Unwin-Hyman. 298 p.

[11] de Ruiter DJ, Berger LR (2000) Leopards as taphonomic agents in dolomitic caves implications for bone accumulations in the hominid-bearing deposits of South Africa. J Archaeol Sci 27: 665–684.

[12] Simons JW (1966) The presence of leopard and a study of the food debris in the leopard lairs of the Mount Suswa caves, Kenya. Bulletin of the Cave Exploration Group of East Africa 1: 51–69.

[13] Sutcliffe AJ (1973) Caves of the east African Rift Valley. Transactions of the Cave Research Group of Great Britain 15: 41–65.

[14] Domínguez-Rodrigo M, Egeland CP, Pickering TR (2007) Equifinality in carnivore tooth marks and the extended concept of archaeological palimpsests: implications for models of passive scavenging in early hominids. In (Pickering, TR., Schick, K. & Toth, N., eds.) Breathing Life into Fossils: Taphonomic Studies in Honor of CK. (Bob) Brain. Bloomington (IN), Stone Age Institute Press. pp. 255–267.

[15] Domínguez-Rodrigo M, Pickering TR (2010) A multivariate approach for discriminating bone accumulations created by Spotted hyenas and leopards: Harnessing actualistic data from East and Southern Africa. . Journal of Taphonomy. 8 (2–3): 155–179.

[16] Pickering TR, Domínguez-Rodrigo M, Egeland CP, Brain CK (2004) Beyond leopards: tooth marks and the contribution of multiple carnivore taxa to the accumulation of the Swartkrans Member 3 fossil assemblage. J Hum Evol: 46 (5): 595–604. 10.1016/j.jhevol.2004.03.002 15120267

[17] Brain CK (1970) New finds at the Swartkrans australopithecine site. Nature 225: 1112–1119. 5418243 10.1038/2251112a0

[18] Brain CK (1993) A taphonomic overview of the Swartkrans fossil assemblages. In: Brain, CK. (Ed.), Swartkrans: A Cave’s Chronicle of Early Man. Transvaal Museum, Pretoria. pp. 257–264.

[19] Sauqué V, Cuenca-Bescós G (2013) The Iberian Peninsula, the last european refugium of *Panthera pardus* Linnaeus 1758 during the Upper Pleistocene. Quaternaire: 24, (1): 2013, p. 35–48.

[20] Blasco F (1997) "In the Pursuit of Game: The Mousterian Cave Site of Gabasa I in the Spanish Pyrennees". J Anthropol Res: 53 (2): 177–217.

[21] Utrilla P, Montes L, Blasco M-F, Torres T, Ortiz J (2010) La Cueva de Gabasa revisada 15 años después: un cubil para las hienas y un cazadero para los Neandertales. Zona Arqueológica 13: 376–389.

[22] Rivals F, Testu A, Moigne AM, Lumley H (2006) The Middle Pleistocene Argali (*Ovis ammon antiqua*) assemblages at the Caune de l’Arago (Tautavel, Pyrénées-Orientales, France): were prehistoric hunters or Carnivores responsible for their accumulation? Int J Osteoarcheol: 249–268.

[23] Testu A, Moigne AM, Lumley H (2011) La panthère *Panthera pardus* des niveaux inférieurs de la Caune de l’Arago à Tautavel (Pyrénées-Orientales, France) dans le contexte des Felidae (Felinae, Pantherinae) de taille moyenne du Pléistocène Européen. Quaternaire: Hors-série (4): 271–281.

[24] Diedrich C (2013) Late Pleistocene leopards across Europe - northernmost European German population, highest elevated records in the Swiss Alps, complete skeletons in the Bosnia Herzegowina Dinarids and comparison to the Ice Age cave art. Quat Sci Rev 76: 167–193.25.

[25] Yravedra J (2006) Acumulaciones biológicas en yacimientos arqueológicos: Amalda VII y Esquilleu III-IV. Trabajos de Prehistoria: 62 (2): 55–78.

[26] Yravedra J (2010) A taphonomic perspective on the origins of the faunal remains from Amalda Cave (Spain). Journal of Taphonomy 8(4): 301–334.

[27] Gisbert M, Pastor M (2009) *Cuevas y Simas de la provincia de Zaragoza*. CEA, Zaragoza. 479 p.

[28] Cuenca-Bescós G, Rofes J, López-García JM, Blain HA, De Marfa RJ, et al (2010) Biochronology of Spanish Quaternary small vertebrate faunas.Quat Int: 212. (2): 109–119.

[29] Pales L, Lambert C (1971) Atlas ostéologique pour servir à la identification des mammifères du quaternaire. Bordeaux. CNRS

[30] Walker R (1985) A guide to post-cranial bones of East African animals, Hylochoerus Press, Norwich. 285 p.

[31] Torres T (1988) Osos (Mammalia, Carnivora, Ursidae) del Pleistoceno de la Península Ibérica. Publicaciones especiales del Boletín Geológico y Minero de España, Special Publication 99: 1–314.

[32] Fernandez H (2001) Ostéologie comparée des petites ruminants eurasiatiques sauvages et domestiques (genres *Rupicapra, Ovis, Capra* et *Capreolus*): diagnose différentielle du squelette appendiculaire Université de Geneva, Facultat de Ciencies. 440 p.

[33] Eisenmann V (1986) Comparative osteology of modern and fossil horses, half-asses, and asses,. In Meadow, RH, Uerpmann H-P. (Eds.), Equids in the Ancient World, Volume I. Dr. Ludwig Reichert Verlag, Wiesbaden. pp. 67–116.

[34] Lyman RL (1994) Vertebrate Taphonomy, Cambridge University Press, Cambridge. 524 p.

[35] Bunn HT (1986) Patterns of skeletal representation and hominids subsistence activities at Olduvai Gorge, Tanzania, and Koobi Fora, Kenya. J Hum Evol 15: 673–690.

[36] Díez JC, Fernández-Jalvo Y, Rosell J, Cáceres I (1999) Zooarchaeology and taphonomy of Aurora Stratum (Gran Dolina, Sierra de Atapuerca, Spain). J Hum Evol: 37 (3 4): 623–652. 10.1006/jhev.1999.0346 10497002

[37] Hillson S (1992) Mammal Bones and Teeth: An Introductory Guide to Methods of Identification. Institute of Archaeology, University College London, London.

[38] Morris P (1978) The use of teeth for estimating the age of wild mammals. En: Butler, PM. y Joysey, KA. (Eds.) Development, function and evolution of teeth. Academic Press. London. pp.483–494.

[39] Morris P (1972) A review of mammalian age determination methods. Mamm Rev 2: 69–104.

[40] Pérez-Ripoll M (1988) Estudio de la secuencia del desgaste de los molares de la *Capra pyrenaica* de yacimientos prehistóricos. Archivo de Prehistoria Levantina XVIII: 83–127.

[41] Vigal CR, Machordom A (1988) Évaluation de la méthode de détermination de l’age en fonction de la mase du cristallin chez le bouquetin (*Capra pyrenaica* Schinz, 1838). Can J Zool 66: 2836–2839.

[42] Tomé C, Vigne J (2003) Roe deer (*Capreolus capreolus*) age at death estimates: New methods and modern reference data for tooth eruption and wear, and for epiphyseal fusion. – Achaeofauna 12: 157–173.

[43] Pérez-Barbería FJ (1994) Determination of age in Cantabrian chamois (*Rupicapra pyrenaica parva*) from jaw tooth-row eruption and wear. J Zool 233: 649–656.

[44] Aitken RJ (1975) Cementum layers and tooth wear as criteria for ageing Roe deer (*Capreolus capreolus*). J Zool 175: 15–28.

[45] Mariezkurrena K (1983) Contribución al conocimiento del desarrollo de la dentición y el esqueleto postcraneal de *Cervus elaphus* . Munibe 35: 149–202.

[46] Azorit C, Analla M, Carrasco R, Calvo JA, Muñoz-Cobo J (2002) Teeth eruption pattern in red deer (*Cervus elaphus hispanicus*) in southern Spain. Anales de Biología 24: 107–114.

[47] d’Errico F, Vanhaeren M (2002) Criteria for identifying red deer (*Cervus elaphus*) age and sex from upper canines: application to the study of Upper Palaeolithic and Mesolithic ornaments. J Archaeol Sci 29: 211–232.

[48] Capaldo SD, Blumenschine RJ (1994) A quantitative diagnosis of notches made by hammerstones percussion and carnivore gnawing on bovid long bones. . Am Antiq:. 59 (4): 724–748.

[49] Pickering TR, Egeland CP (2006) Experimental patterns of hammerstone percussion damage on bones: implications for inferences of carcass processing by humans.J Archaeol Sci. 33: 459–469.

[50] Binford LR (1981) Bones: Ancient Men and Modern Myths. Academic Press, New York. 320 p.

[51] Potts R, Shipman P (1981) Cutmarks made by stone tools on bones from Olduvai Gorge, Tanzania. Nature 291: 577–580.

[52] Shipman P (1983) Early hominid livestyle: hunting and gathering or foraging and scavenging? In: Clutton-Brock, J., Grigson, C. (Eds.), Animals and Archaeology Hunters and Their Prey, vol. 1,. Oxford, BAR International Series 163. pp. 31–49.

[53] Shipman P, Rose J (1983) Early hominid hunting, butchering and carcass-processing behaviors: approaches to the fossil record. J Anthropol Archaeol 2: 57–98.

[54] Shipman P, Fisher DC, Rose J (1984) Mastodon butchery: microscopic evidence of carcass processing and bone tool use. Paleobiology: 10 (3): 358–365.

[55] Haynes G (1980) Evidence of carnivore gnawing on Pleistocene and Recent mammalian bones. Paleobiology: 6 (3): 341–351.

[56] Haynes G (1983) A guide for diferentiating mammalian carnivore taxa responsable for gnaw damage to herbivore limb bones. Paleobiology: 9 (2): 164–172.

[57] Sala N (2012) Tafonomía de yacimientos kársticos de carnívoros en el Pleistoceno. Departamento de Paleontología. Universidad Complutense de Madrid, Madrid, Unpublished Ph. D. 751 p.

[58] Delaney-Rivera C, Plummer TW, Hodgson JA, Forrest F, Hertel F, et al (2009) Pits and pitfalls: taxonomic variability and patterning in tooth mark dimensions. J Archaeol Sci 36: 2597–2608.

[59] Domínguez-Rodrigo M, Piqueras A (2003) The use of tooth pits to identify carnivore taxa in toothmarked archaeofaunas and their relevance to reconstruct hominid carcass processing behaviours. J Archaeol Sci 30: 1385–1391.

[60] Saladié P, Huguet R, Díez C, Rodríguez-Hidalgo A, Carbonell E (2011) Taphonomic modifications produced by modern brown bears (*Ursus arctos*). Int J Osteoarchaeol: doi:10.1002/oa.1237.

[61] Rabal-Garcés R, Cuenca- Bescós G, Canudo JI, Torres T (2012) Was the European cave bear an occasional scavenger? Lethaia:45 (1): 96–108.

[62] Rabal-Garcés R (2013) Estudio paleontológico de *Ursus spelaeus* Rosenmüller, 1794 del Pleistoceno Superior de Coro Tracito (Tella, Huesca, España). Tesis Doctoral, Universidad de Zaragoza, 516 p.

[63] Villa P, Mahieu E (1991) Breakage patterns of human long bones. J Hum Evol 21: 27–48.

[64] Cruz-Uribe K (1991) Distinguishing Hyena from Hominid Bone Accumulations. J Field Archeol: 18 (4): 467–486.

[65] Pickering TR (2002) Reconsideration of criteria for differentiating faunal assemblages accumulated by hyenas and hominids. Int J Osteoarchaeol: 12 (2): 127–141.

[66] Kuhn BF, Berger LR, Skinner JD (2010) Examining Criteria for Identifying and Differentiating Fossil Faunal Assemblages Accumulated by Hyaenas and Hominins using Extant Hyaenid Accumulations.Int J Osteoarchaeol. 20(1): 15–35.

[67] Domínguez-Rodrigo M (1994) Dinámica trófica, estrategias de consumo y alteraciones óseas en la sabana africana: resumen de un Proyecto de Investigación Etoarqueológico (1991- 1993). Trabajos de Prehistoria: 51 (1): 15–37.

[68] Blasco R, Rosell J, van der Made J, Rodríguez J, Campeny G, et al (2011) Hiding to eat: the role of carnivores in the early Middle Pleistocene from the TD8 level of Gran Dolina (Sierra de Atapuerca, Burgos, Spain) J. Arch Sci 38: 3373–3386.

[69] Sala N, Arsuaga JL (2013) Taphonomic studies with wild brown bears (*Ursus arctos*) in the mountains of northern Spain. J.Arch Sci 40: 1389–1396.

[70] Damuth J (1982) Analysis of the preservation of community structure in assemblages of fossil mammals. Paleobiology 8: 434–446.

[71] Noe-Nygaard N, Price TD, Hede SU (2005) Diet of aurochs and early cattle in southern Scandinavia: evidence from 15N and 13C stable isotopes. J Archaeol Sci: 32, 855–871.

[72] Nowak RM (1999) Mammals of the World, 6th edition. Johns Hopkins Univ. Press, Baltimore, MD.1936p.

[73] Burke A, Eisenmann V, Ambler GK (2003) The systematic position of *Equus hydruntinus*, an extinct species of Pleistocene equid. Quat Res 59: 459–469.

[74] Delpech F, Prat F (1980) Les grands mammifères du Sud-Ouest de la France. In Problèmes de stratigraphie quaternaire en France et dans les pays limitrophes, Bulletin de l'Association française pour l'étude du quaternaire. . Supplement. Paris, suppl., n.s. 1: 268–297.

[75] Carranza J, Hidalgo de Trucios SJ, Medina R, Valencia J, Delgado J (1991) Space use by red deer in a Mediterranean ecosystem as determined by radio-tracking. Appl Anim Behav Sci 30(3–4): 363–371.

[76] Mateos-Quesada P (2000) El corzo ibérico. Fundamentos para una particularidad biológica. Trofeo 376: 124–128.

[77] Mateos-Quesada P (2011) Corzo – *Capreolus capreolus*. En: Enciclopedia Virtual de los Vertebrados Españoles. . Salvador, A., Cassinello, J. (Eds.). Museo Nacional de Ciencias Naturales, Madrid.

[78] Carranza J (2011) Ciervo – *Cervus elaphus*. En: Enciclopedia Virtual de los Vertebrados Españoles. Salvador, A., Cassinello, J. (Eds.). Museo Nacional de Ciencias Naturales, Madrid.

[79] Alados CL, Escós J (1996) Ecología y Comportamiento de La Cabra Montés y Consideraciones para su Gestión. CSIC, Madrid.

[80] Granados JE, Pérez JM, Márquez FJ, Serrano E, Soriguer P, et al (2001) La cabra montés (*Capra pyrenaica*, Schinz, 1838). Galemys: 13 (1): 3–37.

[81] Pérez-Barbería FJ, García-González R, Palacios B (2010) Rebeco – *Rupicapra pyrenaica*. En: Enciclopedia Virtual de los Vertebrados Españoles. Salvador, A., Cassinello, J. (Eds.). Museo Nacional de Ciencias Naturales, Madrid.

[82] Barrientos LM (1997) Los Avatares de una población de 35 lobos esteparios. El lobo en la llanura cerealista castellana. Quercus 139: 14–17.

[83] Mech LD, Boitani L (2003) Wolves: Behaviour, Ecology and Conservation. University of Chicago Press. 472p.

[84] Delibes M (1980) Feeding ecology of the Spanish lynx in the Coto Doñana. Acta Theriol 25: 309–324.

[85] Rodríguez-Hidalgo A, Lloveras L, Moreno-García M, Saladié P, Canals A, et al (2013) Feeding behaviour and taphonomic characterization of non-ingested rabbit remains produced by the Iberian lynx (*Lynx pardinus*) J Archaeol Sci: 40. 7: 3031–3045.

[86] Valdiosera CE, Garcia-Garitagoitia JL, Garcia N, Doadrio I, Thomas M, et al (2008) Surprising migration and population size dynamics in ancient Iberian brown bears (*Ursus arctos*) PNAS. 105: 5123–5128. 10.1073/pnas.0712223105 PMC2278212 18347332

[87] Davison J, Ho SYW, Bray SC, Korsten M, Tammeleht E, et al (2011) Late-Quaternary biogeographic scenarios for the brown bear (*Ursus arctos*), a wild mammal model species Quat Sci Rev. 30: 418–430.

[88] Clevenger A, Purroy FJ (1991) Ecologia del Oso pardo en Espana. Monografıas M.N.C.N. (CSIC). 155 p.

[89] Elosegui MM (2009) El oso pardo en los Pirineos. Lynx edicions. 286p.

[90] Arsuaga JL, Baquedano E, Pérez-González A, Sala N, Quam RM, et al (2012) Understanding the ancient habitats of the last-interglacial (late MIS 5) Neanderthals of central Iberia: paleoenvironmental and taphonomic evidence from the Cueva del Camino (Spain) site. Quat Int 275: 55–75.

[91] Sala NMT, Arsuaga JL, Laplana C, Zapata BM, García MJG, et al (2011) Un paisaje de la Meseta durante el Pleistoceno Superior. Aspectos paleontológicos de la Cueva de la Zarzamora (Segovia, España). Bol R Soc Esp Hist Nat Sec Geol: 105 (1–4): 67–85.

[92] Cáceres I (2002) Tafonomía de yacimientos antrópicos en Karst. Complejo Galería (Sierra de Atapuerca, Burgos), Vanguard Cave (Gibraltar) y Abric Romaní (Capellades, Barcelona). Ph.D. dissertation, Universitat Rovira i Virgili. 661p.

[93] Stiner MC, Arsebuk G, Howell FC (1996) Cave bears and Paleolithic artifacts in Yarimburgaz Cave: dissecting a palimpsest. Geoarchaeol: 11 (4): 279–327.

[94] Voorhies M (1969) Taphonomy and population dynamics of an early Pliocene vertebrate fauna, Knox County, Nebraska. Contrib. Geol., Spec. Pap. No. 1. Univ. Wyo. Press; Laramie, Wyoming. 69p.

[95] Coard R (1999) One bone, two bones, wet bones, dry bones: transport potentials under experimental conditions. J Archaeol Sci 26: 1369–1375.

[96] Lyman RL (1984) Bone density and differential survivorship of fossil classes. J Anthropol Archaeol 3: 259–299.

[97] Huguet R, Arsuaga JL, Pérez-González A, Arriaza MC, Sala-Burgos MTN, et al (2010) Homínidos y hienas en el Calvero de la Higuera (Pinilla del Valle, Madrid) durante el Pleistoceno Superior. Resultados preliminares. Zona Arqueológica 13: 444–458.

[98] Salazar-García DC, Power RC, Sanchis-Serra A, Villaverde V, Walker MJ, et al.. (2013) Neanderthal diets in central and southeastern Mediterranean Iberia,Quat Int, in press

[99] Villa P, Soressi M (2000) Stone tools in carnivore sites: the case of Bois Roche1. J Anthropol Res 56: 187–215.

[100] Marchal F, Monchot H, Coussot C, Desclaux E, Deschamp P, et al (2009) Neandertals paleoenvironment in Western Provence: The contribution of Les Auzières 2 (Méthamis, Vaucluse, France). Comptes. Rendus. Palevol 8: 493–502.

[101] Villa P, Sánchez-Goñi MF, Cuenca-Bescós G, Grün R, Ajas A, et al (2010) The archaeology and paleoenvironment of an Upper Pleistocene hyena den: An integrated approach. J Archaeol Sci: 37 (5): 919–935.

[102] Monchot H (2005) Un assemblage original au Paléolithique moyen : le repaire à hyènes, porcs-épics et hominidés de la grotte Geula (Mont Carmel, Israël). Paléorient: 31 (2): 27–42.

[103] Monchot H, Aouraghe H (2009) Deciphering the taphonomic history of an Upper Paleolithic faunal assemblage from Zouhrah Cave/El Harhoura 1, Morocco. Quaternaire: 20 (2): 239–253.

[104] Villa P, Castel JCH, Beauval C, Bourdillat V, Golberg P (2004) Human and carnivore sites in the European Middle and Upper Paleolithic: similarities and differences in bone modification and fragmentation. Rev. Paleobiol: 23 2: 705–730.

[105] Domínguez-Rodrigo M, Barba R (2006) New estimates of tooth mark and percussion mark frequencies at the FLK Zinj site: the carnivore-hominidcarnivore hypothesis falsified. JHum Evol 50: 170–194. 16413934 10.1016/j.jhevol.2005.09.005

[106] Curio E (1976) The Ethology of Predation. Springer Verlag, Berlin. 250p.

[107] Domínguez-Rodrigo M (1994) Las razones adaptativas del comportamiento subsistencial de los animales carnívoros y sus estrategias iniciales de consumo de presas: Relevancia en el proceder tafonómico. Cuaderno de Prehistoria Castellana 16: 9–17.

[108] Mills MGL, Harvey M (2001) African predators. Cape Town: Struik Publishers

[109] García N, Arsuaga JL (2001) Les Carnivores (Mammalia) des sites du Pléistocène ancien et moyen d’Atapuerca (Espagne) L’Anthropologie. 105: 83–93.

[110] Barroso Ruiz C, Botella Ortega D, Caparros M, Moigne AM, Celiberti V, et al (2011) The Cueva del Angel (Lucena, Spain): an Acheulean hunters habitat in the south of the Iberian peninsula. Quat Int 243: 105–26.

[111] Quam R, Arsuaga JL, Bermúdez De Castro JM, Díez JC, et al (2001) Human remains from Valdegoba Cave (Huérmeces,Burgos, Spain). J Hum Evol 41: 385–435. 11681860 10.1006/jhev.2001.0486

[112] Villaluenga A (2009) Yacimientos del Pleistoceno superior en la Península Ibérica con presencia de restos de oso. Munibe 60: 17–33.

[113] Kurten B (1976) The Cave Bear story: Life and death of a vanished anima, Columbia University Press, New York. 163p.

[114] Rogers LL (1981) A bear in its lair. Natural History: 90 (10): 64–70.

[115] Rogers LL (1987) Effects of food supply and kinship on social behavior, movements, and population growth of black bears in northeastern Minnesota. J Wildl Manage: 51 (2): 1–72.

[116] Stiner MC (1998) Mortality analysis of Pleistocene bears and its paleoanthropological relevance. J Hum Evol 34: 303–326. 9547458 10.1006/jhev.1997.0198

[117] Naves J, Fernández-Gil A, Rodríguez C, Delibes M (2006) Brown bear food habits at the border of its range: a long-term study. J Mammal: 87 (5): 899–908.

[118] Stiner MC (1999) Cave bear ecology and interactions with pleistocene humans. Ursus 11: 41–58.

[119] Pinto Llona AC, Andrews P, Etxebarría F (2005) Tafonomía y paleoecología de úrsidos cuaternarios cantábricos. Fundación Oso de Asturias, Asturias. 679p.

[120] Carrión JS, Riquelme JA, Navarro C, Munuera M (2001) Pollen in hyaena coprolites reflects late glacial landscape in southern Spain. Palaeogeogr Palaeoclimatol, Palaeoecol: 176 (1–4): 193–205.

[121] Mills G, Hofer H (1998) *Hyaenas. Status Survey and Conservation Action Plan*. IUCN, Gland, Switzerland, and Cambridge, UK. IUCN/SSC Hyaena Specialist Group.

[122] Sutcliffe AJ (1970) Spotted Hyaena: Crusher, Gnawer, Digester and Collector of Bones. Nature 227: 1110–1113. 5451104 10.1038/2271110a0

[123] Kruuk H (1972) The spotted hyena: a study of predation and social behavior. The University Chicago Press. 335p.

[124] Diedrich C (2009) Steppe lion remains imported by Ice Age spotted hyenas into the Late Pleistocene Perick Caves hyena den in Northern Germany. Quat Res: 71 (3): 361–374.

[125] Cooper SM, Holekamp KE, Smale L (1999) “A seasonal feast: long-term analysis of feeding behaviour in the spotted hyaena (*Crocuta crocuta*),”. Afr J Ecol: 37 2: 149–160.

[126] Hayward MW (2006) Prey preferences of the spotted hyaena *Crocuta crocuta* and evidence of dietary competition with lion *Panthera leo* . J Zool: 270 4: 606–614.

[127] Fosse P, Avery G, Fourvel JB, Lesur-Gebremariam J, Monchot H, et al (2010) Los cubiles actuales de hiena: síntesis crítica de sus características tafonómicas a partir de la excavación de nuevos yacimientos (República de Djibuti, África del Sur) y la información publicada. Zona arqueológica 13: 108–117.

[128] Altuna J (1972) Fauna de mamíferos de los yacimientos prehistóricos de Guipúzcoa, con catálogo de los mamíferos cuaternarios del Cantábrico y del Pirineo occidental. Munibe: 24 (1–4): 1–464.

[129] Falguères C, Yokoyama Y, Arrizabalaga A (2005) La Geocronología del yacimiento pleistocénico de Lezetxiki (Arrasate, País Vasco). Crítica de las dataciones existentes y algunas nuevas aportaciones. Munibe. Antropología-Arkeología 57: 93–106.

[130] Clot A (1980) La Grotte de la Carrière (Gerde, Hautes-Pyrénées). Stratigraphie et paléontologie des Carnivores. Thèse de Doctorat, Université Paul Sabatier, Toulouse, 502p.

[131] Castaños P (1990) Los carnívoros de los yacimientos prehistóricos vascos. Munibe. Antropología-Arkeología 42: 253–258.

[132] Bertram BCB (1999) Leopard. In D.W. Macdonald (ed.), The encyclopedia of mammals. Andromeda Oxford, Oxford.pp. 44–48.

[133] Hayward MW, Henschel P, O'Brien J, Hofmeyr M, Balme G, et al (2006) Prey preferences of the leopard (*Panthera pardus*). J Zoo: 270 2: 1–16.

[134] Steyn V, Funston PJ (2006) A case of cannibalism in leopards. S Afr J Wildl Res: 36 2: 189.

[135] García N, Arsuaga JL (1998) The carnivore remains from hominid-bearing Trinchera- Galería, Sierra de Atapuerca, Middle Pleistocene site (Spain). Geobios: 31 (5): 659–674.

[136] Esteban-Nadal M (2012) Can Archaeozoology and Taphonomy contribute to knowledge of the feeding habits of the Iberian wolf? J Archaeol Sci: 39 10: 3208–3216.

[137] Okarma H (1997) Der Wolf. Ökologie, Verhalten, Schutz. Parey Verlag, Berlin. pp. 1–160.

[138] Jêdrzejewska B, Jêdrzejewski W (1998) Predation in vertebrate communities,Springer Verlag, Berlin. 450p.

[139] Ansorge H, Kluth G, Hahne S (2006) Feeding ecology of wolves *Canis lupus* returning to Gemany. Acta Theriol 51(1): 99–106.

[140] Yravedra J, Lagos L, Bárcena F (2011) A taphonomic study of wild wolf (Canis lupus) modification of horse bones in Northwestern Spain. Journal of Taphonomy: 9 (1): 37–65.

[141] Castel JC, Coumont MP, Boudadi-Maligne M, Prucca A (2010) Rôle et origine des grands carnivores dans les accumulations naturelles. Le cas des loups (*Canis lupus*) de l’Igue du Gral (Sauliac-sur-Célé, Lot, France). Rev. Paléobiologie: 29 (2): 411–425.

[142] Castel JC (2004) L'influence des canidés sur la formation des ensembles archéologiques. Caractérisation des destructions dues a loup. Rev. Paléobiologie: 23 (2): 675–693.

[143] Stiner MC (2004) Comparative ecology and taphonomy of spotted hyenas, humans, and wolves in Pleistocene Italy. Rev. Paléobiologie: 23 (2): 771–785.

[144] Montoya P, Alberdi MT, Blázquez AM, Barbadillo LJ, Fumanal MP, et al (1999) La fauna del Pleistoceno Inferior de la Sierra de Quibas (Abanilla, Murcia). Est. Geol 55: 127–161.

[145] Pons-Moyà J (1983) Presencia de *Lynx spelaea* (Fissipeda, Mammalia) en el Pleistoceno inferior de la Península Ibérica. Paleontologia i Evolució 18: 39–42.

[146] Altuna J (1980) Hallazgo de un lince nórdico (*Lynx lynx* L. Mammalia) en la sima de Pagolusieta, Gorbea (Vizcaya). Munibe 32: 317–322.

[147] Altuna J (1981) Restos óseos del yacimiento prehistórico de Rascaño. El Paleolítico Superior de la Cueva de Rascaño (Gonzalez Echegaray, J. y Barandiaran, J. eds.), C.I.M.A. 3, Santander: 223–269.

[148] Gil-Sánchez JM, Ballesteros-Duperón E, Bueno-Segura JF (2006) Feeding ecology of the Iberian lynx *Lynx pardinus* in eastern Sierra Morena (Southern Spain). Acta Theriol 51: 85–90.

[149] Fosse P (1997) Variabilité des assemblages osseux créés par l’hyène des caverns. Paleo 9: 15–54.

[150] Diedrich C (2011) The *Crocuta crocuta spelaea* (Goldfuss, 1823) population and its prey from the late Pleistocene Teufelskammer cave hyena den besides the famous Paleolithic Neanderthal cave (NRW, NW Germany). Hist Biol: 23 (2–3): 237–270.

[151] Diedrich C (2011) The Late Pleistocene spotted hyena *Crocuta crocuta spelaea* (Goldfuss, 1823) population from the Zoolithen Cave at Gailenreuth (Bavaria, South Germany) e a hyena cub raising den of specialized cave bear scavengers in Boreal Forest environments of Central Europe. His Biol: 23 (4): 335–367.

[152] Diedrich C (2011) Periodical use of the Balve Cave (NW Germany) as a Late Pleistocene Crocuta crocuta spelaea (Goldfuss, 1823) den: hyena occupations and bone accumulations vs. human Middle Palaeolithic activity. Quat Int 233: 171–184.

[153] Diedrich C (2012) The Ice Age spotted *Crocuta crocuta spelaea* (Goldfuss, 1823) population, their excrements and prey from the Late Pleistocene hyena den Sloup Cave in the Moravian Karst; Czech Republic. His Biol: 24 (2): 161–185.

[154] Diedrich C (2012) Late Pleistocene Crocuta crocuta spelaea (Goldfuss, 1823) clans as prezewalski horse hunters and woolly rhinoceros scavengers at the open air commuting den and contemporary Neanderthal camp site Westeregeln (central Germany). J. Archaeol Sci: 39 (6): 1749–1767.

[155] Diedrich C (2012) Europe’s first Upper Pleistocene *Crocuta crocuta spelaea* (Goldfuss, 1823) skeleton from the Koneprusy Caves e a hyena cave prey depot site in the Bohemian Karst (Czech Republic). His Biol: 24 (1): 63–89.

[156] Fernández Rodríguez C, Ramil RP, Martínez Cortizas A (1995) Characterization and depositional evolution of hyaena (*Crocuta crocuta*) coprolites from La Valiña cave (northwest Spain). J Archaeol Sci 22: 597–607.

[157] Davis S (2002) The mammals and birds from the Gruta do Caldeirão. Portugal. Rev. port. arqueol: 5 (2): 29–98.

[158] Blasco F, Montes L (1997) "Los hiénidos del yacimiento Musteriense de Gabasa 1 (Huesca, España)". Bolskan 14: 9–27.

[159] Arrizabalaga A, Altuna J (2000) Labeko Koba (País Vasco). Hienas y humanos en el Paleolítico Superior. Munibe 52: 193–343.

[160] Jordá JF, Maestro A, Aura JE, Álvarez-Fernández E, Avezuela B, et al (2011) Evolución paleogeográfica, paleoclimática y paleoambiental de la costa meridional de la Península Ibérica durante el Pleistoceno superior. El caso de la Cueva de Nerja (Málaga, Andalucía, España) Bol R Soc Esp Hist Nat 105: 137–147.

[161] Daura J, Sanz M, García N, Allué E, Vaquero M, et al (2013) Terrasses de la Riera dels Canyars (Barcelona, Spain): the landscape of Heinrich Event 4 north of the “Ebro frontier”. and implications for modern human dispersal into Iberia, Quaternary Sci Rev 60: 26–48.

[162] Prendergast M, Domínguez-Rodrigo M (2008) Taphonomic analyses of a hyena den and a natural-death assemblage near Lake Eyasi (Tanzania). Journal of Taphonomy 6: 301–335.

[163] Blumenschine RJ (1986) Carcass consumption sequences and the archaeological distinction of scavenging and hunting. J Hum Evol:15 (8): 639–659.

[164] Mitchell BL, Shenton JB, Uys JCM (1965) Predation on large mammals in the Kafue National Park, Zambia. Zoologica Africana 1: 297–318.

[165] FitzGibbon CD, Fanschawe JH (1989) The condition and age of Thomson’s gazelles killed by cheetahs and wild dogs. J Zool 218: 99–107.

[166] Fitzgibbon CD, Lazarus J (1995) Antipredator behavior of Serengeti ungulates. In: Serengeti II: Dynamics, Conservation and Management of an Ecosystem (Ed. by A. R. E. Sinclair & P. Arcese), Chicago: University of Chicago Press. pp. 274–296.

[167] Blumenschine R, Marean CW (1993) A carnivore's view of archaeological bone Assemblages. En: Hudson, J. (Ed.) From bones to behavior. Ethnoarchaeological and experimental contributions to the interpretation of faunal remains. Illinois, Center of Archaeological Investigations. Southern Illinois University press. pp. 273–300.

[168] Marean CW, Kim SY (1998) Mousterian largemammal remains from Kobeh Cave: Behavioral implications for Neanderthals and early modern humans. Curr Anthropol: 39 (Supplement): 79–113.

[169] Selvaggio MM (1994) Identifying the Timing and Sequence of Hominid and Carnivore Involvement with Plio-Pleistocene Bone Assemblages from Carnivore Tooth Marks and Stone-Tool Butchery Marks on Bone Surfaces. Ph.D. dissertation, Rutgers University.

[170] Selvaggio MM, Wilder J (2001) Identifying the involvement of multiple carnivore taxa with archaeological bone assemblages. J Archaeol Sci 28: 465–470.

[171] Saladié P (2009) Mossegades d’omnívors. Aproximació experimental i aplicació zooarqueològica als jaciments de la Sierra de Atapuerca. Tesis Doctoral, Universitat Rovira i Virgili, Tarragona, 940p.

